# Microwave-assisted synthesis, antioxidant activity, docking simulation, and DFT analysis of different heterocyclic compounds

**DOI:** 10.1038/s41598-023-31995-w

**Published:** 2023-03-27

**Authors:** Mona A. Shalaby, Asmaa M. Fahim, Sameh A. Rizk

**Affiliations:** 1 Chemistry Department, Faculty of Science, Ain Shams University, Abbassia, P.O. 11566, Cairo, Egypt; 2 Green Chemistry Department, National Research Centre Dokki, P.O. Box 12622, Cairo, Egypt

**Keywords:** Chemistry, Green chemistry, Organic chemistry, Theoretical chemistry

## Abstract

In this investigation, pressure microwave irradiation was used to clarify the activity of 1-(2-hydroxyphenyl)-3-(4-methylphenyl)prop-2-en-1-one (3) towards several active methylene derivatives utilized the pressurized microwave irradiation as green energy resource . Chalcone **3** was allowed to react with ethyl cyanoacetate, acetylacetone, and thioglycolic acid; respectively, at 70 °C with pressure under microwave reaction condition to afford the corresponding 2-hydroxyphenylcyanopyridone, 2-hydroxyphenyl acetylcyclohexanone, and thieno[2,3-*c*]chromen-4-one derivatives respectively. Moreover, the reaction of chalcone **3** with hydrogen peroxide with stirring affords the corresponding chromen-4-one derivative. All the synthesized compounds were confirmed through spectral tools such as FT-IR, ^1^HNMR, ^13^CNMR, and mass spectrum. Furthermore, the synthesized heterocycles were exhibited excellent antioxidant activity and comparable with vitamin C, where the presence of the OH group increases the scavenger radical inhibition. Furthermore, the biological activity of compound **12** was demonstrated through molecular docking stimulation using two proteins, **PDBID:** 1DH2 and **PDBID:** 3RP8, which showed that compound **12** possesses greater binding energy and a shorter bond length comparable with ascorbic acid. Also, the compounds were optimized through DFT/B3LYP/6-31G (d,p) basis set and identification of their physical descriptors, whereas the compound **12** was confirmed through X-Ray single structure with Hirsh field analysis of the compound to know the hydrogen electrostatic bond interaction, and correlated with the optimized structure by comparing their bond length, bond angle, FT-IR, and NMR, which gave excellent correlation.

## Introduction

**Figure 1:**
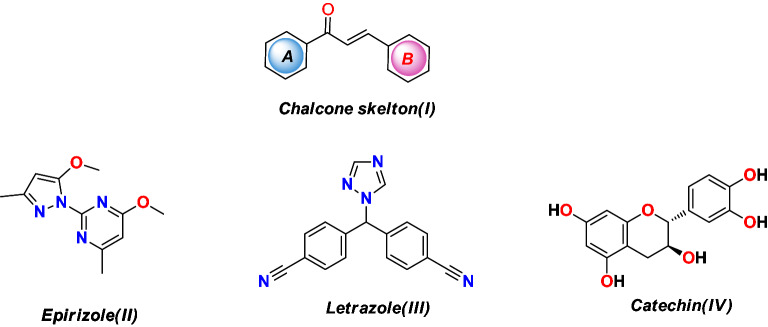
Structure of chalcones and different drugs contains different heterocyclic rings.

**Figure 2:**
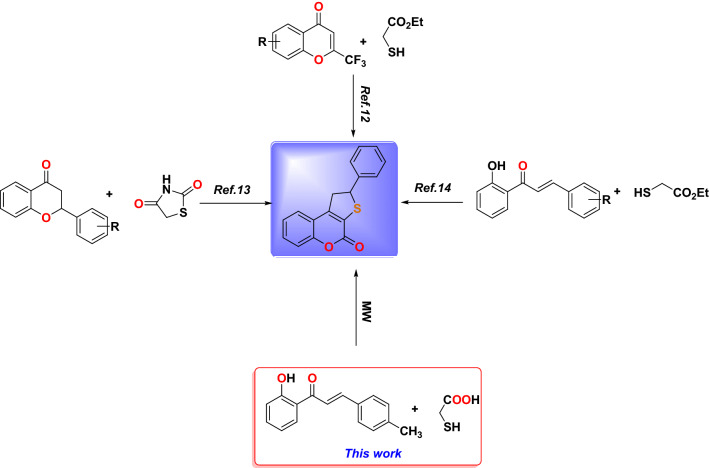
Strategies and synthetic routes used for the synthesis of 1,2-dihydrothieno[2,3-*c*]chromen-4-one.

Chalcones are important compounds found in nature or as synthetic analogs that are essential intermediates for the synthesis of different flavonoids and isoflavonoids and are also used in different biological evaluations and medicinal chemistry^1–5^.This activity is due to the chemical flexibility and twist of the rings as displayed in Fig. 1(I), which gave it the ability to synthesize different biological heterocyclic rings such as pyrazole, cyanopyridine, flavanones, and di-aryl cyclohexanones^6–8^ such as the Epirizole(**II**), which is a nonsteroidal anti-inflammatory drug^9^, while the Letrazole drug(**III**), which used as aromatase inhibitor after breast cancer surgery^10,11^, also the flavanoid Catechin (**IV**) antioxidant drug from plants^12^ as shown in Fig. 1**.** Various methods were reported to synthesize 1,2-dihydrothieno[2,3-*c*]chromen-4-one derivatives, as shown in Fig. 2^13–15^.

Furthermore, the use of green tools in chemical reactions takes most scientists' interest, from these tools the use of microwave heating emerged as the most reliable of activated compounds, which enhanced their reaction time, enhancing conversion, and increased selectivity^6,9,16–18^. Moreover, chemically, radical scavengers play an important role in biology, chemistry, and materials science, for example, they are used in food storage, pharmaceuticals, cosmetics, petroleum products, oils, and rubber, as well as for electronic devices^19,20^ and development of new radical scavengers with applications in industry and pharmaceuticals has become increasingly important^21–25^. Moreover, the docking stimulation enhanced and confirmed the biological studies of these chemical reactions^26–28^.

In this study, we synthesized various heterocycles from the reaction of 1-(2-hydroxyphenyl)-3-(*p*-tolyl)prop-2-en-1-one (**3**) with different methylene compounds utilizing microwave irradiation to give the cyanopyridine, cyclohexanone and chromen-4-one derivatives, which confirmed and investigated their antioxidant activity. These compounds showed excellent oxidative behavior due to the presence of the OH group, which increases the scavenger radical inhibition and these results were confirmed through molecular docking with the most binding energy. Furthermore, all synthesized compounds were optimized through DFT/B3LYP/6-31G(d,p) basis set and showed the stability of them due to the high band energy gap, also compound **12** was confirmed through single-X-ray and showed compatible with the theoretical results through bond length, angles correlation, and FT-IR, and NMR analysis.

## Results and discussion

### Chemistry

**Figure 3:**
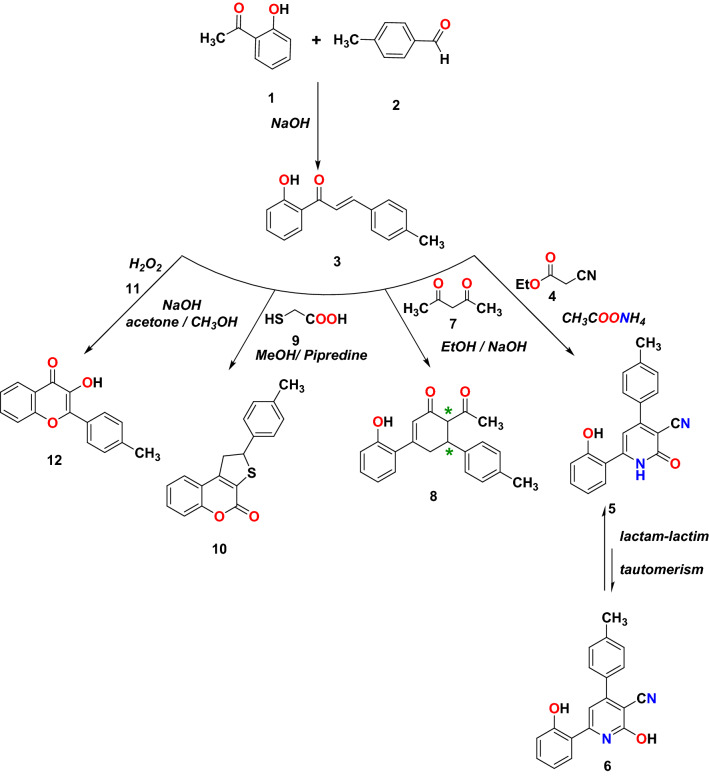
Synthesis of different heterocylic compounds.

**Figure 4:**
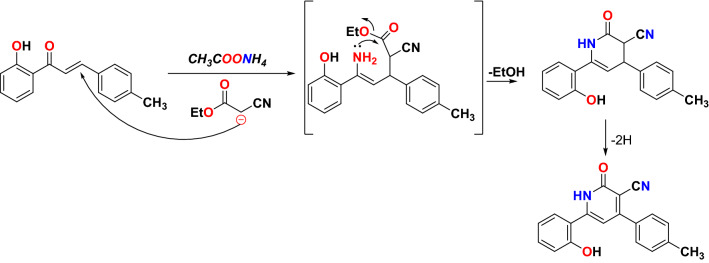
Plausible mechanism of formation compound **5**.

The reactivity of chalcones towards nucleophiles is due to the conjugation between the carbonyl group and the double bond. Consequently, the nucleophiles can attack both carbonyl and double bonds and giving an interesting wide range of cyclized compounds^29^. The 2′-hydroxychalcone derivative **3** was synthesized using the method described previously^30^. The microwave-assisted reactions of chalcone **3** with different active methylene, such as ethyl cyanoacetate **4**, acetylacetone **7**, and thioglycolic acid **9**, are outlined in Fig. 3, in addition to its reaction with hydrogen peroxide **11**. First, the reaction of chalcone **3** with ethyl cyanoacetate **4** in the presence of ammonium acetate afford the corresponding cyanopyridine **5**. The FT-IR of the resulting compound **5** showed different bands at 3174, 2217, and 1637 cm^−1^ due to NH, C≡N, and C=O groups; respectively. Furthermore, the ^1^H NMR spectrum showed broad singlet peaks at δ 10.52 and 12.38 ppm due to NH (lactam) and OH (phenol); respectively. This indicates the presence of compound **5** in lactam form only, as shown in Fig. 3. A plausible mechanism for this reaction is illustrated in Fig. 4.

**Figure 5:**
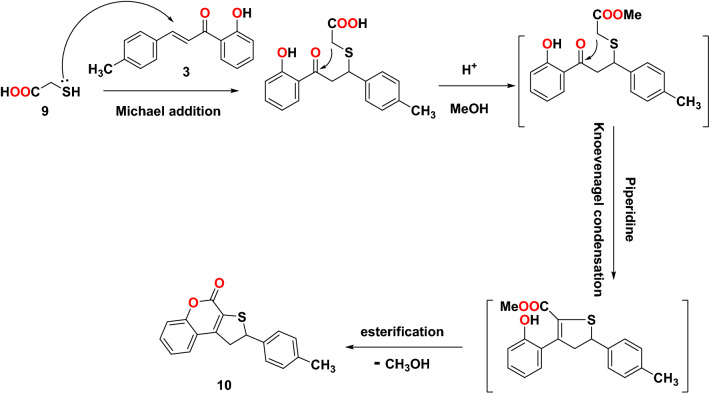
A possible mechanism for the formation of compound **10**.

Next, the Michael addition reaction of chalcone **3** with acetylacetone **7** in presence of NaOH as a base followed by internal Claisen condensation gave the corresponding cyclohexanone **8**. Two chirality centers at C-5 and C-6 formed in the resulted compound **8** from this cyclo-condensation. Unfortunately, the reaction was not stereoselective as both configurations of chiral carbon centers were expected to result a mixture of diastereoisomers. No attempt was taken to separate the diastereomeric structures and were characterized as a mixture. The IR spectra of compound **8** showed bands at 3107, 1721, and 1625 due to aromatic C-H, and CO, C=C groups; respectively. Moreover, the ^1^H NMR for compound **8** showed the characteristic peak of the vinylic proton of the cyclohexanone ring at 6.39 ppm. The two H-4 protons are non-equivalent protons, they appeared at 2.84 ppm as multiplet peaks, while the H-5 appeared at 3.62 ppm as a multiplet. The H-6 proton appeared as a doublet at 4.24 ppm. The aromatic protons appeared between 6.74 and 7.30 ppm. The D2O exchangeable singlet peak of OH appeared at 9.92 ppm. Also, ^13^C NMR supported the structure of compound **8** where two signals appeared at 196.32 and 205.97 ppm due to the two carbonyl groups and a signal at 135.06 ppm due to vinylic carbon C-2. The mass spectrum exhibited a peak at *m/z* 320 attributable to the molecular ion as displayed in Fig. 3. Then, the dihydrothienocoumarin **10** was obtained by the Michael addition reaction of thioglycolic acid **9** in methanol and piperidine. IR spectra of compound **10** showed bands at 3028, 1711, and 1601 cm^−1^ due to aromatic C–H, C=O, and C=C groups, respectively. Furthermore, the ^1^H NMR confirmed the structure skeleton, where the ABX systems due to the three protons of dihydrothienyl ring. The signals appeared at δ 3.72 and 4.01 ppm with *J* = 18 Hz for HA and HB, resonated as a pair of doublets of doublets, respectively. The HX in the dihydrothienyl ring appeared as a triplet at δ 5.36 ppm with *J* = 6.4 Hz. All the other aromatic protons were observed with their expected chemical shifts. The mass spectrum exhibited a peak at *m/z* 293 attributable to the molecular ion as displayed in Fig. 3. A plausible mechanism for this reaction is illustrated in Fig. 5. Thioglycolic acid** 9** started the Michael addition reaction by attacking the chalcone compound **3** to produce an adduct that form the ester adduct then undergoes Knoevenagel condensation reaction to produce methyl 3-(2-hydroxyphenyl)-5-(*p*-tolyl)-4,5-dihydrothiophene-2-carboxylate. In the end, this compound is converted into coumarin compound **10** through intramolecular esterification.

**Figure 6:**
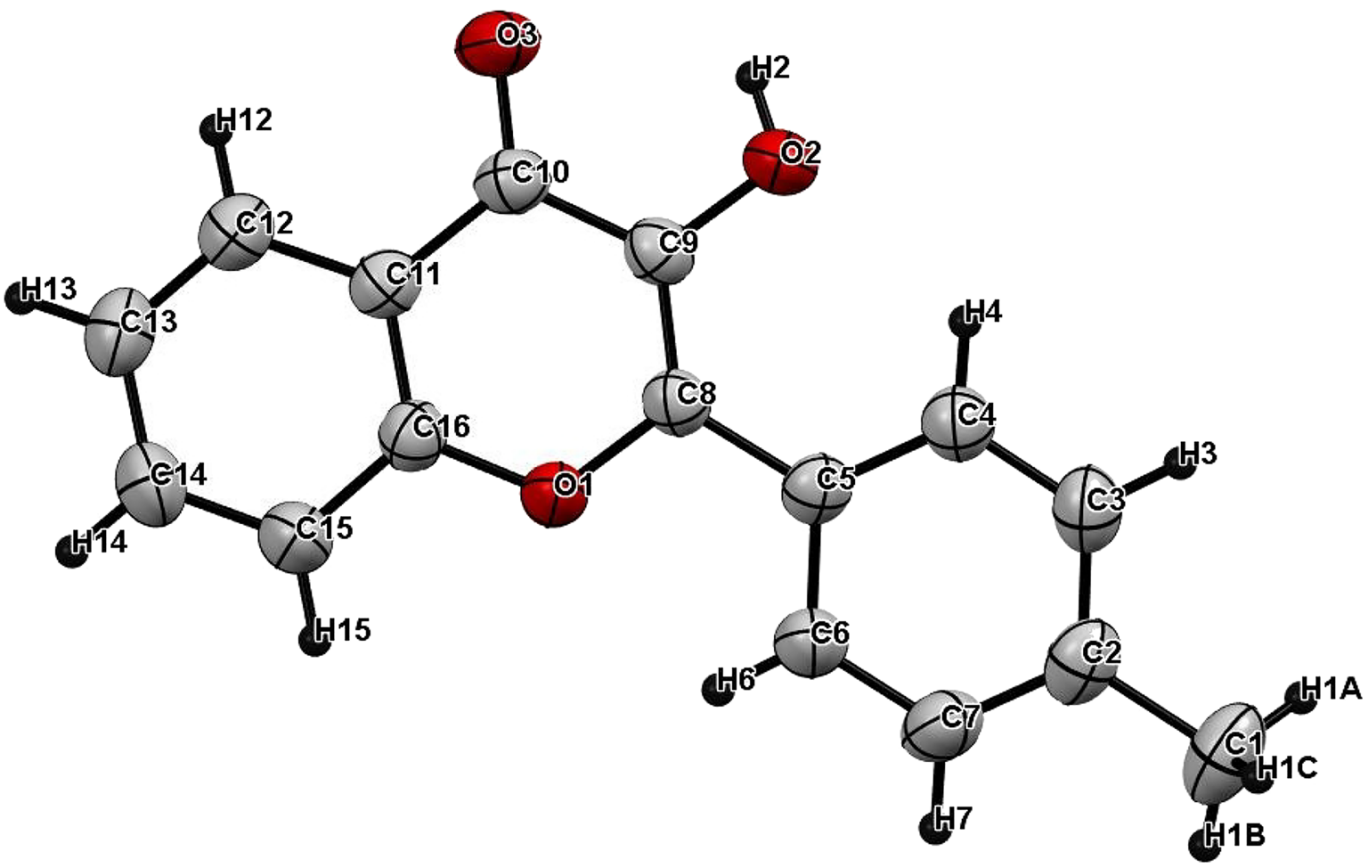
X-ray crystal structure of **12.**

Finally, the reaction of chalcone **3** with sodium hydroxide/hydrogen peroxide-mediated in acetone and methanol as a solvent formed the C-4′ substituted flavonol **12**, as shown in Fig. 2. This reaction is known as the Algar-Flynn-Oyamada reaction, in which chalcones undergo an oxidative cyclization to form flavonols^31^. The IR spectra of compound **12** showed bands at 3284, 3107, and 1607 cm^−1^ due to OH, aromatic C–H, and C=O groups; respectively. The region between 7.3 and 8.2 ppm in the ^1^H NMR (DMSO-*d6*) spectrum indicates the presence of 8 aromatic protons. The ^13^C NMR spectra of flavonol **12** show the C-4 signal at 173.02 ppm and the C-3 signal at 145.37 ppm. Compound **12** was further confirmed by single crystal X-ray, as shown in Fig. 6.

## Biological action

### Antioxidant activity

**Figure 7:**
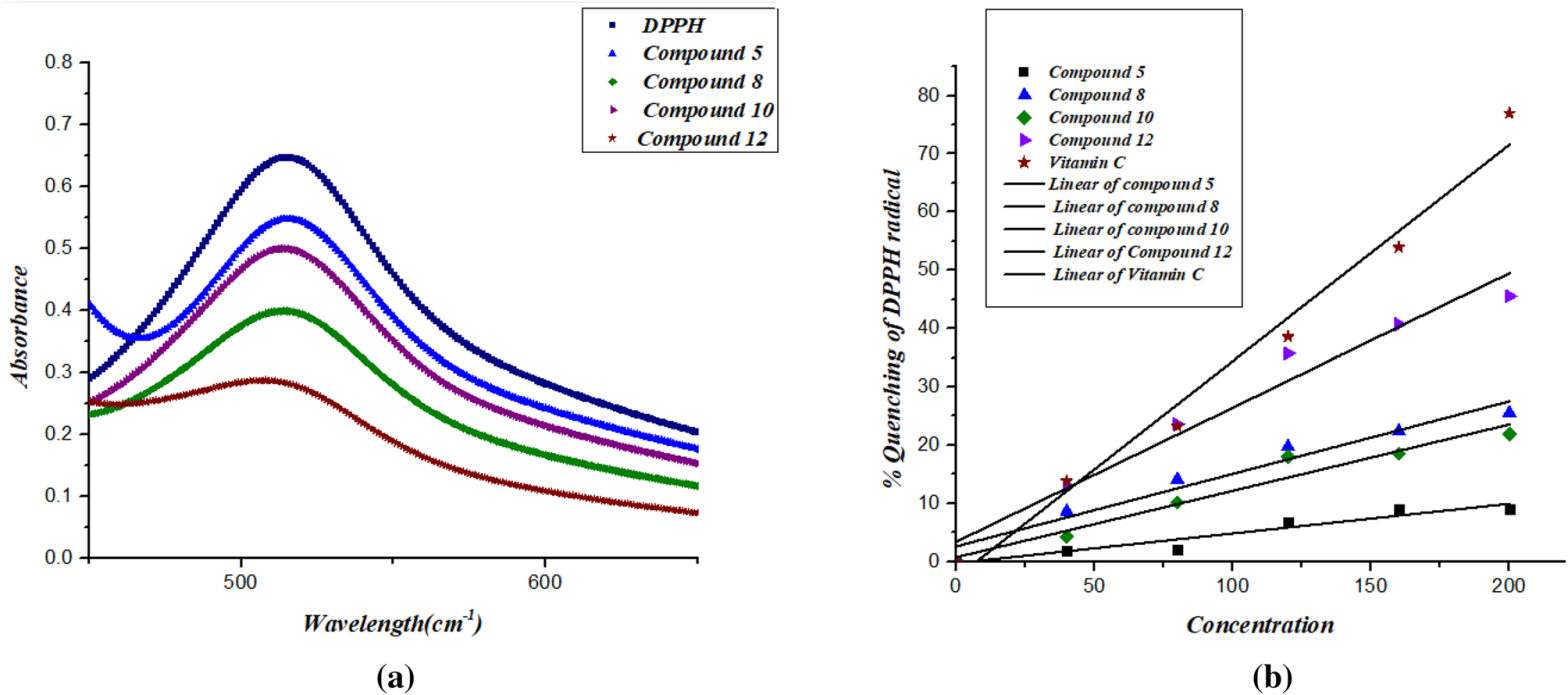
**(a)** Typical absorption spectra of 100 µM of the DPPH radical alone and in presence of a 200 µM concentration of compounds **5–12** and vitamin C. **(b)** A diagram of the percentage of quenching of the DPPH radical against the concentration of the antioxidants **5–12** and vitamin C.

Compounds **5**, **8**, **10**, and **12** were investigated for their radical scavenging abilities in methanol by using the DPPH assay, in which DPPH radicals change from purple to yellow when quenched by an antioxidant. DPPH radicals are generally monitored at 517 nm (Fig. 7a), at which the absorption decreases with antioxidants, and Fig. 7b shows the percentage inhibition as a function of antioxidant concentration. Table 1 indicates that all compounds, except compound **5**, possess antioxidant properties. Among all synthesized compounds, compound **12** showed the best antioxidant properties, exhibiting an IC50 of approximately 202.20 µM and being comparable to vitamin C (IC50 at 141.9 µM). The IC50 values of the remaining compounds are decrease to approach the value of vitamin C in the order of **8** > **10**. The order of the property depends on the radical stability formed in the derivatives^32^.

**Table 1:** IC50 values of afforded heterocyclic compounds (**5–12**) for the DPPH radical in methanol.

Compounds	IC50 (µM)
**5**	–
**8**	380.19
**10**	431.10
**12**	202.20
**Vitamin C**	141.90

### Structure activity relationship (SAR)

**Figure 8:**
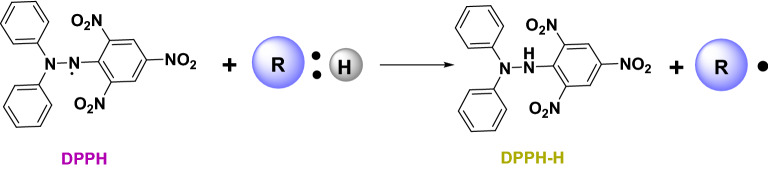
Mechanism of 2,2-diphenyl-1-picrylhydrazyl (DPPH) reaction with antioxidant, where R: H is aradical scavenger; R. is a radical scavenger.

The structure–activity relationship (SAR) strategy seeks to establish correlations between the biological activity of investigated substances and their chemical structure. When the odd electron in the DPPH assay is linked with a hydrogen or electron-donating antioxidant, as illustrated in Fig. 8, the odd electron's high absorption band at 519 nm disappears. In general, phenolic compounds exhibit good antioxidant activity because DPPH produces stable phenoxide radicals by abstracting hydrogen atoms. However, the free radical scavenging activity of compound **5** shows negligible antioxidant activity. Comparing compounds **10** and **12** which share the structure of the chromene core, compound **12** was found to have more antioxidant properties than compound **10**. On the other hand, comparing compounds **5** and **8** which contain pyridinone and cyclohexenone cores, respectively, we found that compound **8** has antioxidant activity while compound **5** showed no activity. The lowest activity of compound **5** can be attributed to the influence of the electron withdrawing cyano group, while the power of compound **12** as a DPPH radical scavenger can be attributed to its strong hydrogen donor properties^33^.

### Docking stimulation

**Figure 9:**
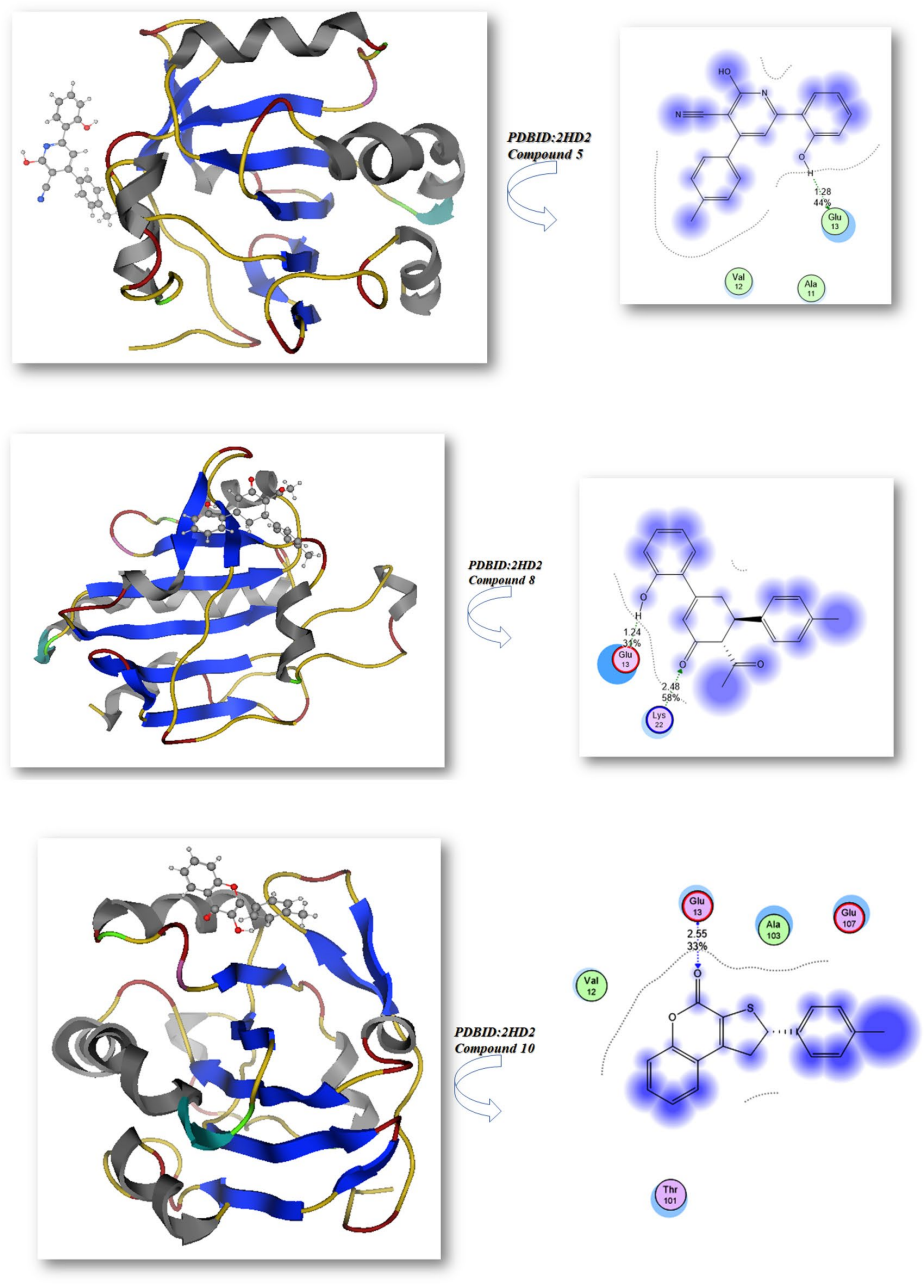

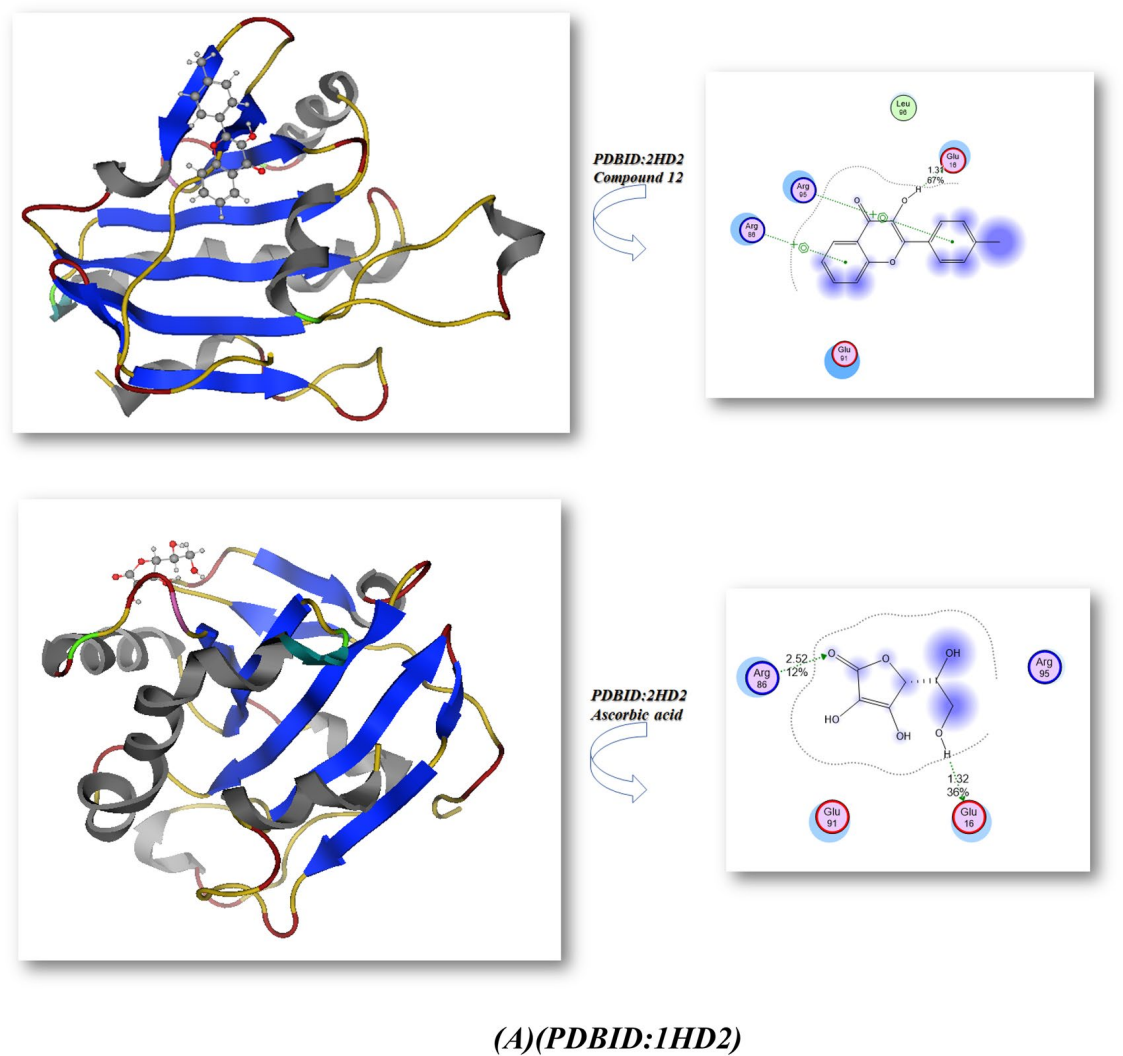

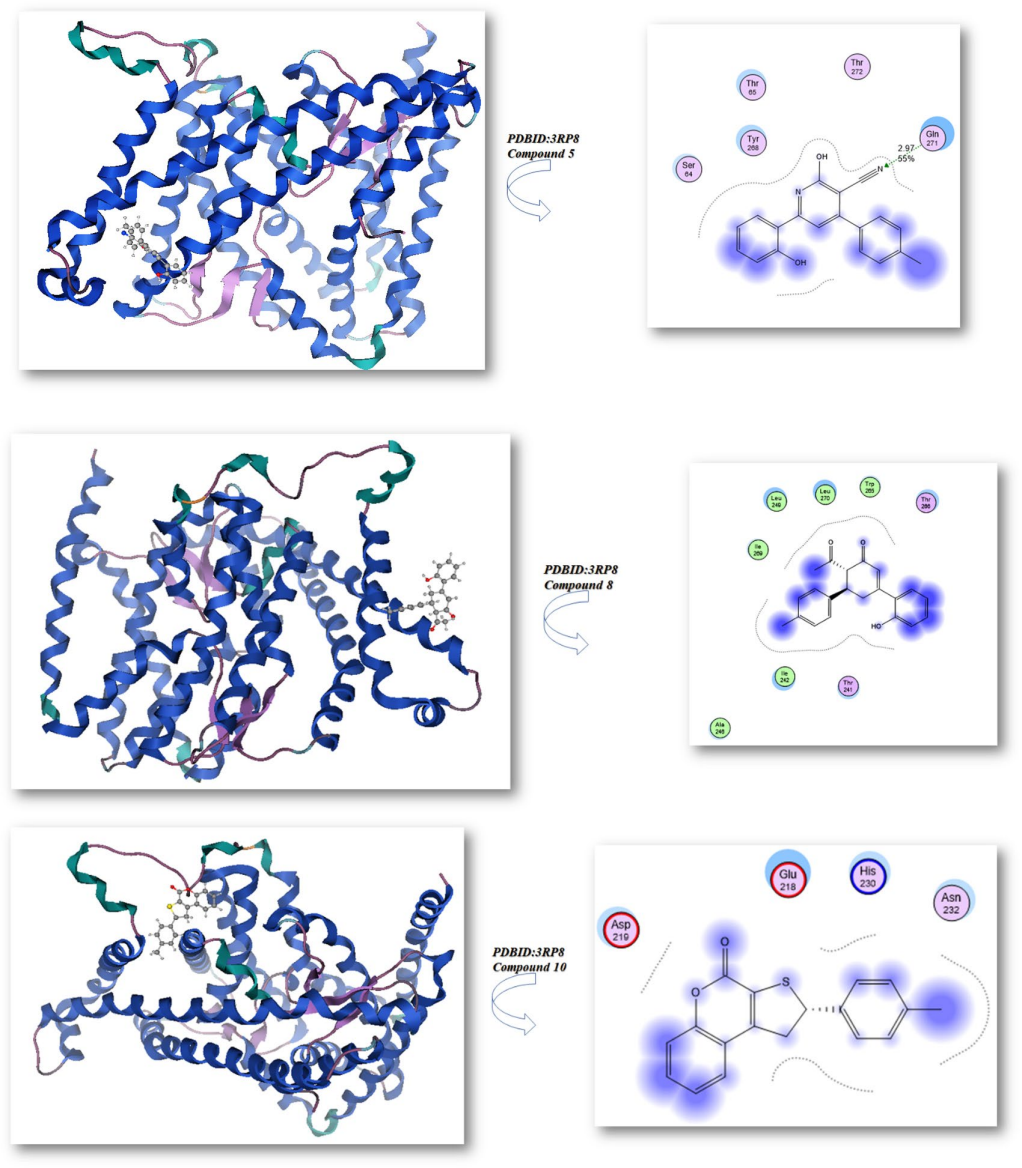

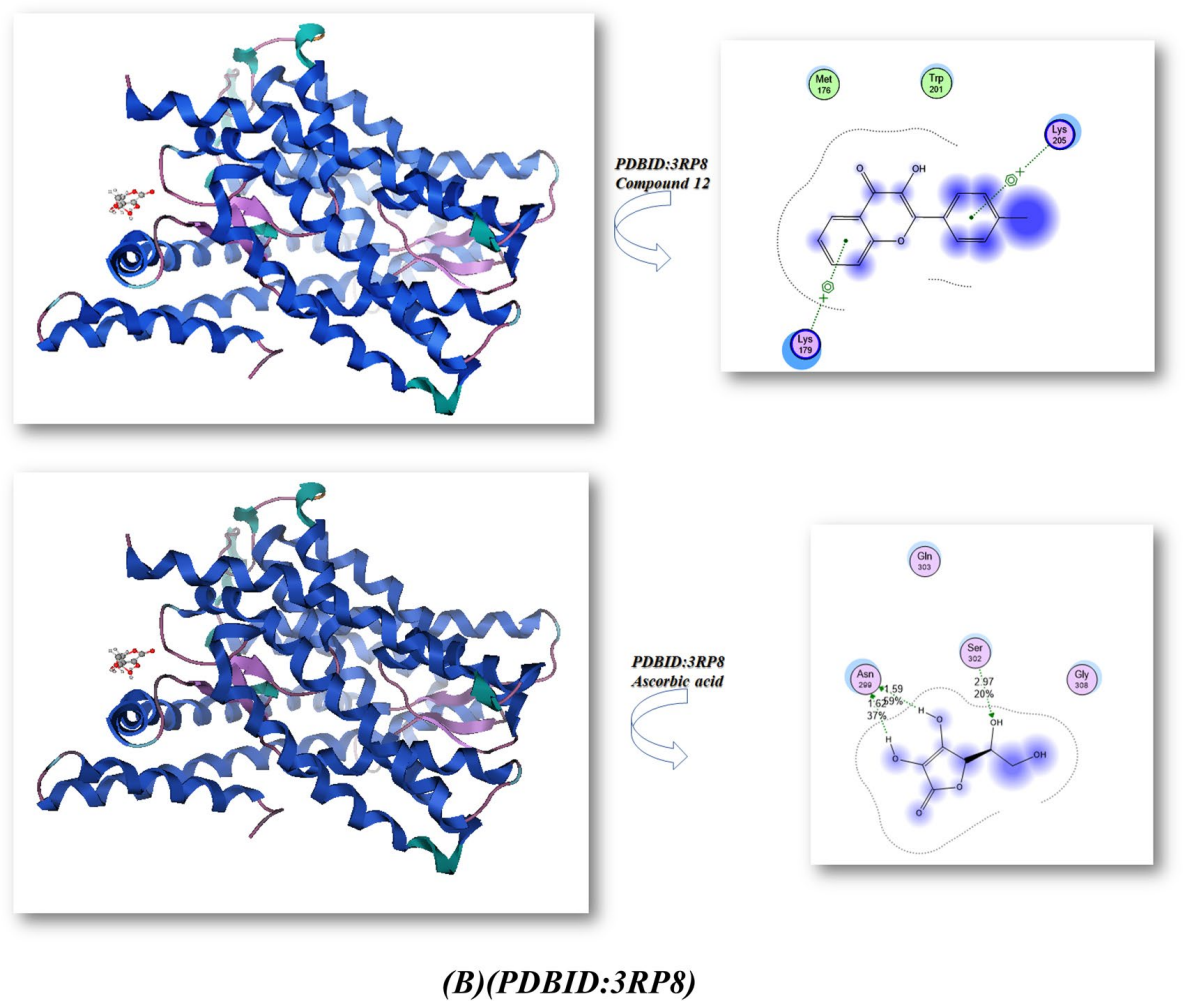
Docking analysis graph of compounds with PDBID(1HD2 and 3RP8); respectively.

Bond lengths in units were added to the complex docking molecular analysis utilizing Moe software 29. Figure 9 and Table 2 show the implementation of the minimization energies to maintain the geometrical optimization and systematic investigations with an RMS gradient of 0.01 of Human peroxiredoxin 5 (PDB ID: 1HD2)30 and the crystal structure of Klebsiella pneumoniae R204Q HpxO complexed with FAD (PDBID: 3RP8)31. From Fig. 9A, it can be seen that the compounds 5, 8, 10, 12, and ascorbic acid had different binding affinities with **PDBID:**1HD2. The compound 12 had the highest binding affinity, with a bond length of 1.31 Å and a variety of amino acids (Glu 16, Arg 95, Leu 96, Arg 80, Glu 91), due to presence of two OH groups which enhanced antioxidant action towards DPPH, while the compound 5 showed less binding affinity due its majority of the attachments with CN, while the ascorbic acid had excellent binding with protein, with a bond length of −9.3 kcal/mol and a Moreover, compounds 8, 10 outstanding efficacy equivalent to ascorbic acid but they are still active. The majority of the attachments to C=O and OH groups were found in ascorbic acid, which has ascorbic acid (−8.2, −8.9, and −8.4 kcal/mol) and bond lengths in the range (1.24–2.55 Ả), which enhances scavenges radical pocket and boosts electrostatic energy. Moreover, Table 2 and Fig. 9 showed that chemicals 5, 8, 10, 12, and ascorbic acid stimulated docking (B). Ascorbic acid exhibits the highest binding energy with protein **PDBID:**3RP8 with an energy affinity of −9.33440 kcal/mol with a length of 1.62 Å and various proteins (Asn 299, Ser 302, Glu 308, Gln 303), whereas compound 12 displayed a binding energy of −8.7 kcal/mol with a length of 2.67 Å, and various amino acids (Mrt 176, Trp 201, Lys 205, Lys 179). Also, Compounds 5, 8, and 10 showed the lowest binding energy with a range of −7.2, −7.6, −7.2 kcal/mol and the least length with 2.9 Å, 2.8 Å, 2.5 Å; respectively so we concluded that the docking results were compatible with experimental results. Furthermore, the presence of more OH groups in the compounds such as compound **12** enhanced the radical scavenge on the pocket of protein, while the presence of compound **5** in lactam form and presence of cyan group in the compound decrease the radical scavenger and not get activity with the proteins.

**Table 2:** The molecular docking metal complexes with PDBID:1HD2 and PDBID:3RP8:

	**(PDB: 1HD2)**				**(PDB: 3RP8)**		
	Affinity of Energy (kcal/mol)	Distance (Å)	Amino acids		Affinity of Energy (kcal/mol)	Distance(Å)	Amino acids
**5**	−8.0	1.28	Glu 13, Ala 11, Val 12	**5**	−7.2	2.97	Gln 271, Thr 272, Thr 65, Tyr 208, Ser64
**8**	−8.2	1.24, 2.48	Lys 22, Glu 13,	**8**	−7.6	2.84	Ile 269, Leu 249, Leu 270, Trp 265, Thr 265,Thr 266, Ile 242, Thr 241, Ala 246
**10**	−8.9	2.55	Glu 13, Ala103, Glu 107, Val 12	**10**	−7.2	2.49	Asp 219, Glu 218, His 230, Asn 232
**12**	−8.9	1.31	Glu 16, Arg 95, Leu 96, Arg 80, Glu 91	**12**	−8.7	2.67	Mrt 176, Trp 201, Lys 205, Lys 179
**Ascorbic acid (vitamin C)**	−9.2	1.32, 2.52	Arg 86, Arg 95, Glu 16, Glu 91	**Ascorbic acid (vitamin C)**	−9.3	1.62, 1.59, 2.97	Asn 299, Ser 302, Glu 308, Gln 303

## Computational investigation

### Physical descriptor’s

**Figure 10:**
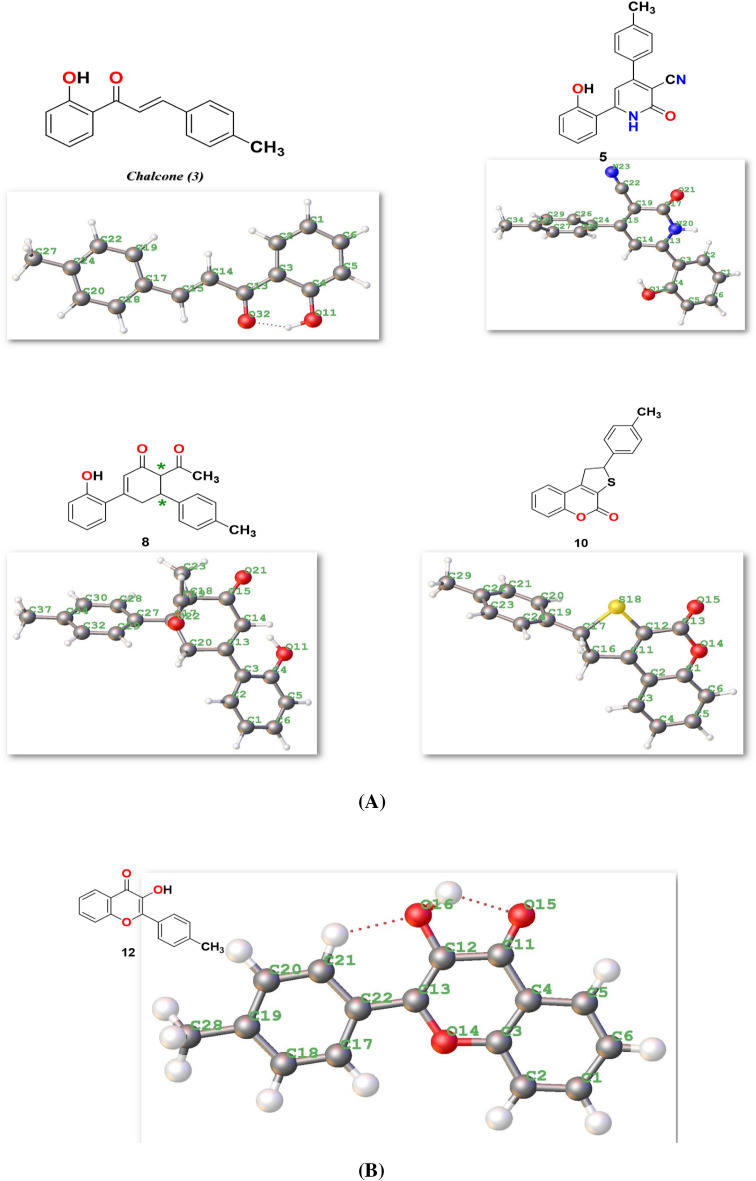
**(A)** Chemical and the optimized structure of compounds **3**, **5**, **8**, and **10** utilized DFT/631(G) basis set. **(B)** Chemical and the optimized structure of compound **12** utilized DFT/631(G) basis set.

The optimization of the desired compounds was investigated utilizing Gaussian(09)34 utilized DFT/B3LYP/6-31 (G) basis set^35,36^ as displayed in Fig. 10 Table 3 lists the physical properties used in the optimization of molecular structures of compounds **3**, **5**, **8**, **10**, and **12** concerning (σ) absolute softness, (χ) electronegativity, (ΔNmax) electronic charge, (η) absolute hardness, (ω) global electrophilicity, (S) global softness, and (Pi) chemical potential, from Eqs. (1–8) estimated with B3LYP/6–31G(d,p). The total energy of the compounds **5**, **8**, **10** and **12** were more stable than the starting chalcone 3 which indicate the stability of them, and also compound **10** showed more stability with energy (−1243.02 au) (−33,824.3132 eV) due to presence of O and S in its structure and increase their electronegativity character. Moreover, the hardness of chemical system which showed the resistance of electron cloud deformation through small perturbations encountered during the chemical process.

**Table 3:** Ground state energies of compounds utilizing DFT/B3LYP/6-31G(d,p) and their physical parameters.

Δ*E* = *E*_*LUMO*_ − *E*_*HOMO*_	(1)	$\chi=\frac{-(E_{HOMO}+E_{LUMO})}{2}$	(2)		
$\eta=\frac{(E_{LUMO}-E_{HOMO})}{2}$	(3)	σ = 1/η	(4)		
Pi = − χ	(5)	S = 1/2 η	(6)		
ω = Pi^2^/2η	(7)	ΔN_max_ = −Pi/η	(8)		

DFT/B3LYP/6-31G(d,p)					
	**3**	**5**	**8**	**10**	**12**
Physical descriptors					
*ET *(au)	−768.61	−992.43	−1037.98	−1243.02	−842.63
*EHOMO *(eV)	−6.13	−6.22	−6.18	−5.79	−5.82
*ELUMO *(eV)	−2.35	−2.31	−2.12	−1.87	−2.01
Δ*E* (eV)	3.77	3.91	4.06	3.92	3.81
*µ* (D)	4.78	7.43	3.09	6.36	3.24
*χ* (eV)	4.24	4.27	4.158	3.83	3.922
*η* (eV)	1.88	1.95	2.03	1.96	1.90
*σ* (eV)	0.52	0.51	0.49	0.50	0.52
*Pi* (eV)	−4.24	−4.27	−4.15	−3.83	−3.92
*S* (eV)	0.264	0.255	0.246	0.254	0.262
*ω* (eV)	4.763	4.665	4.252	3.746	4.037
Δ*N*_*max*_	2.246	2.185	2.046	1.954	2.059

Furthermore, the dipole moment of compound **5** is very high comparable with other compounds and difference in dipole moment between the chalcone and compound **5** with (2.65D) due to presence of C≡N which make easily made separation of charges and get more reactivity^37^.Consequently, unlike hard molecules η (eV), soft molecules' electron densities can be easily altered and indicate the extent resistance of change of electron cloud density in the system, we notice 8 had high values with (2.03 eV) (46.813 kcal/mol) compared with chalone which has (1.88 eV) (≈ 43.354 kcal/mol), which cyclize and reactivity and they are nearest to each other^38,39^.

**Figure 11:**
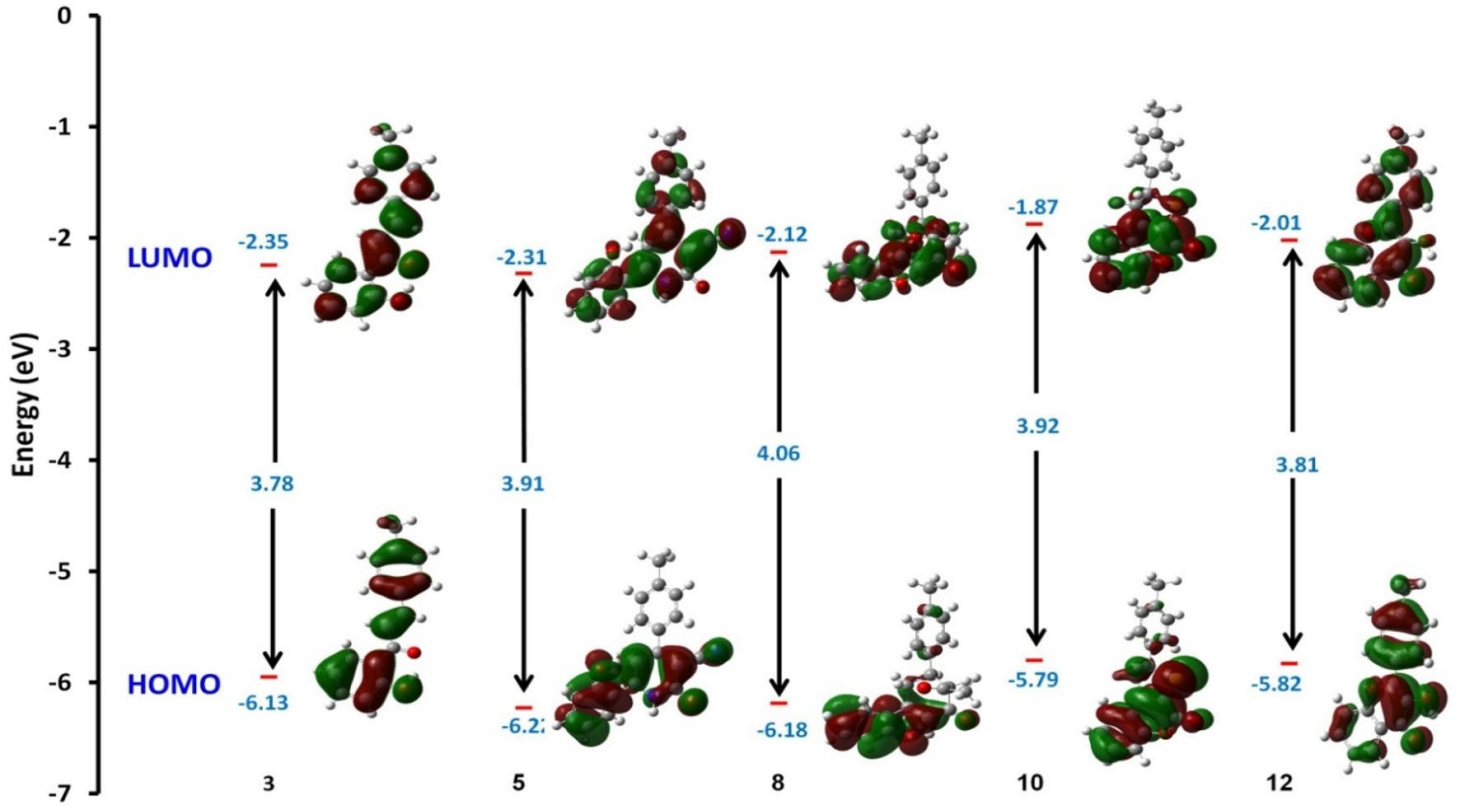
Schematic diagrams of HOMO and LUMO energy levels of compounds **3**, **5**, **8**, **10** and **12** obtained from the DFT calculation with B3LYP/6-31G(d,p).

**Figure 12:**
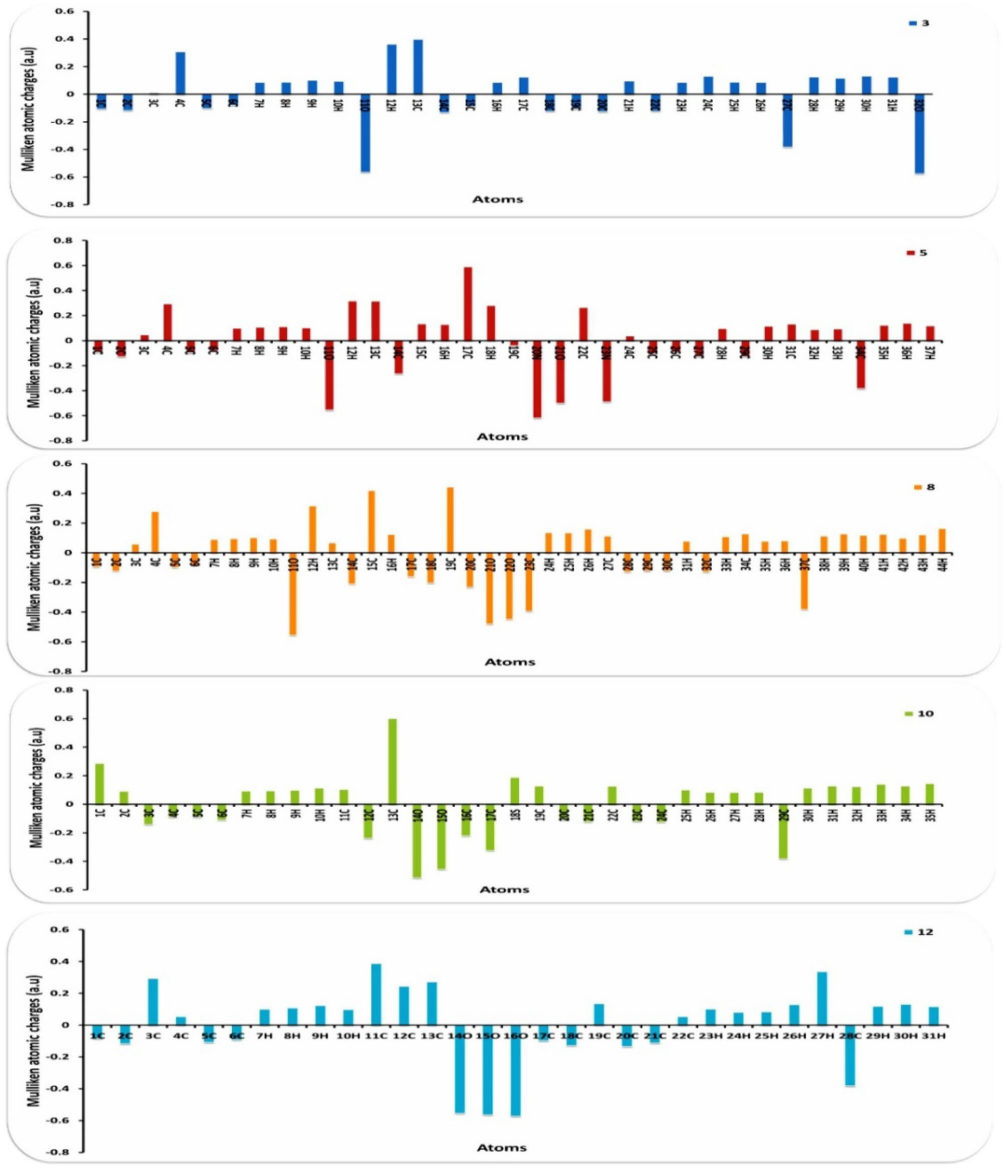
Distribution of calculated Mulliken charges for compounds **3**, **5**, **8**, **10** and **12**.

Furthermore, chemical softness (σ), which describes the ability of an atom or a group of atoms to accept electrons, was noticed from the following Table 3 the value of (σ) were ranging for (0.49–0.52 eV) for all compounds and gave their ability to cyclized and be stable. Also, electrophilicity describes the ability of an electrophile to acquire electronic charge, in addition to its resistance to exchanging electronic charge with its surroundings and the chalcone 3 showed extent value with (4.24 eV) (97.777 kcal/mol) due to presence of =CH− bond which can easily react with different nuclophilies to give stable compounds. In addition to providing information about electron transfer (chemical potential) and stability (hardness), it describes global chemical reactivity better^40,41^. Furthermore, the energy gap (ΔE) between the highest occupied molecular orbital (HOMO) and the lowest unoccupied molecular orbital (LUMO) can be used to assess a molecule's kinetic stability and chemical hardness-softness^42–44^. Molecules with a large HOMO–LUMO gap are hard, while molecules with a small HOMO–LUMO gap are soft. Also, hard molecules usually exhibit high kinetic stability as well as low chemical reactivity, while the contrary is for soft molecules. Moreover the hardness and softness are controlled by its ΔE, where a large value of ΔE indicates high kinetic stability, while a low value indicates high chemical reactivity^45^. According to the results for the chemical reactivity descriptors of compounds **3**, **5**, **8**, and **10**, the high hardness (2.03 eV) and low softness (0.246 eV) values display lower intramolecular charge transfer (Table 3). Also, compound **8** has the highest ΔE among them reflecting its kinetic stability, while compound **3** has the lowest ΔE reflecting its high chemical reactivity. The HOMO–LUMO orbitals and their distributions and energy levels were calculated at the B3LYP/6-31G(d, p) level for all the synthesized heterocyclic compounds, as illustrated in Fig. 11. For the start compound **3**, the HOMO distributes charges over the molecule except for the carbonyl group, while its LUMO distributes charges over the molecule except for the methyl group. For the produced heterocyclic compounds, The HOMO of compound **12** distributes charges over the entire molecule, while the HOMO of the remaining compounds distributes charges over the molecules except for the tolyl group. The charge density of LUMO for compounds **5** and **12** is localized over the whole molecule, while the LUMO of the remaining compounds distributes charges over the molecules except the tolyl group. Additionally, Mulliken atomic charges and electronic populations for these compounds were calculated for each atom using the B3LYP/6-31G(d, p) basis sets. The atomic charges of compounds **3**, **5**, **8**, and **12** are shown in Fig. 12, and their optimized structures with atomic numbering are shown in Fig. 10. Atoms with a lower electronegative attract electrons from those with a higher electronegative in the structure, delocalizing the negative charges in these atoms. The charges of carbon atoms are both positive and negative, as seen in Fig. 12. O and N are electron-withdrawing atoms, so the C atom attached to them has a positive charge. The most acidic hydrogens are phenolic hydrogen (12H) in compounds **3**, **5**, **8**, and **12,** while (35H) for compound **10**.

### Hirshfeld surface analysis

**Figure 13:**
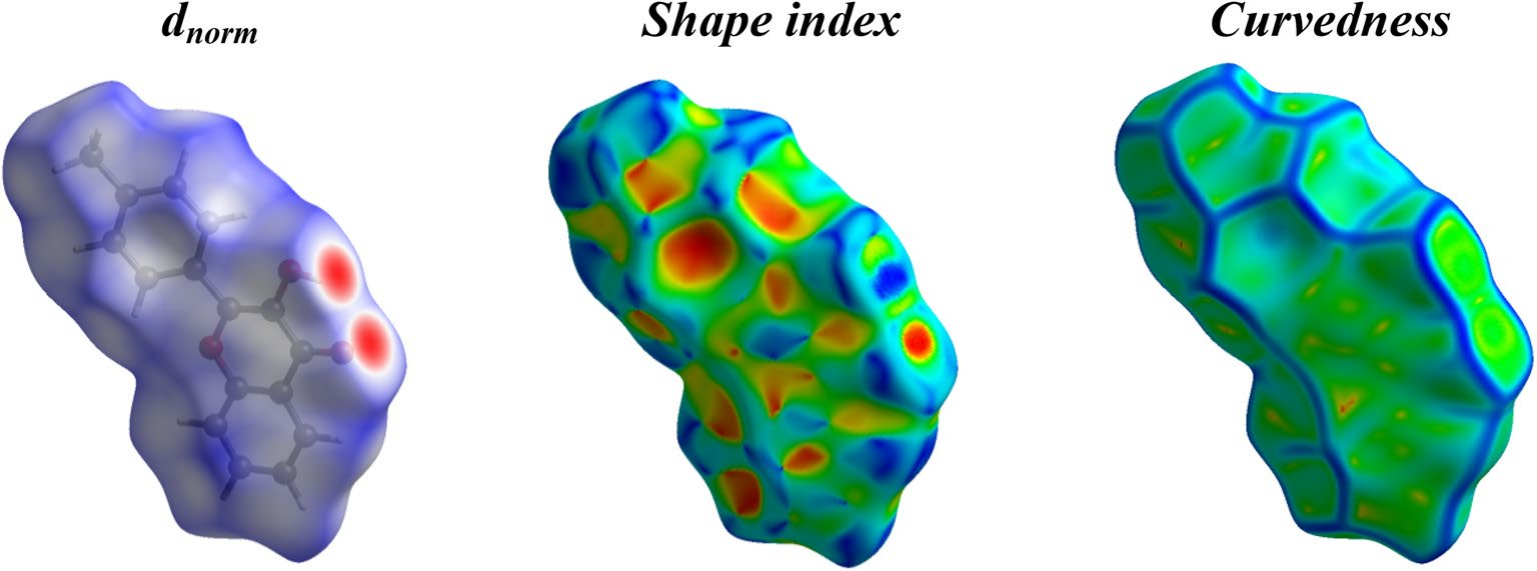
Hirshfeld surfaces (d_norm_, shape index, and curvedness) of compound **12**.

**Figure 14:**
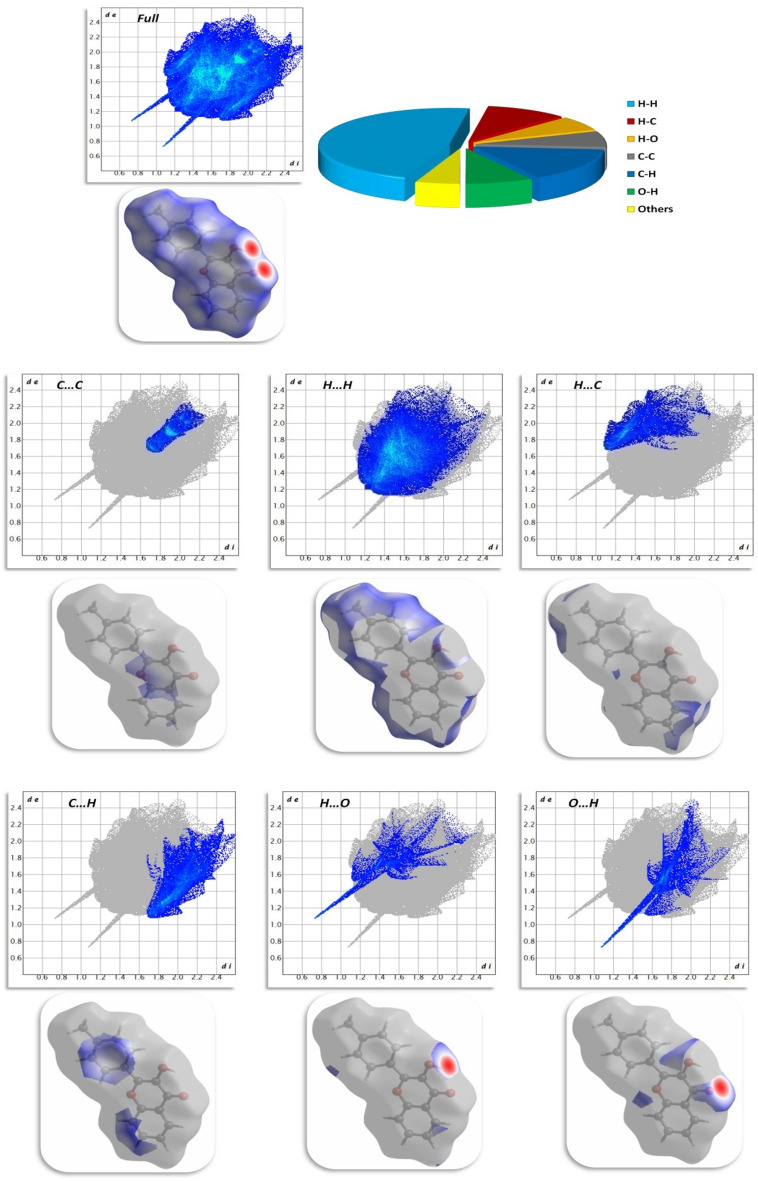
Selected fingerprint plots and d_norm_ surfaces for important interactions in compound **12**.

Hirshfeld surface analysis is an effective way to visualize and understand interactions between molecules in crystals^46–48^. CrystalExplorer 17.5 was used to calculate and visualize the interactions^49^. Figure 13 illustrates Hirshfeld surfaces for compound **12**, and Fig. 14 illustrates the 2D-fingerprint plot detailing all possible interactions in compound **12** as well as the decomposed dnorm maps. The Hirshfeld surface mapped by d_norm_ was coloured in red, white, and blue for contacts whose van der Waals radius was less than, equal to, and greater than the sum of contacts, respectively. The surface contains the dark red circulars that were evidently caused by hydrogen bonding between the O···H/H···O atoms. H…H interactions have the highest percentage of contacts (46.8%) in the Hirshfeld surface, while C…H, H…C, H…O, O…H, and C…C interactions have respective percentages of 15.3%, 10.1%, 6.6%, 8.4%, and 7.1%.

### Comparative study between X-ray single crystal and theoretical studies

#### Bond length and bond angles

**Figure 15:**
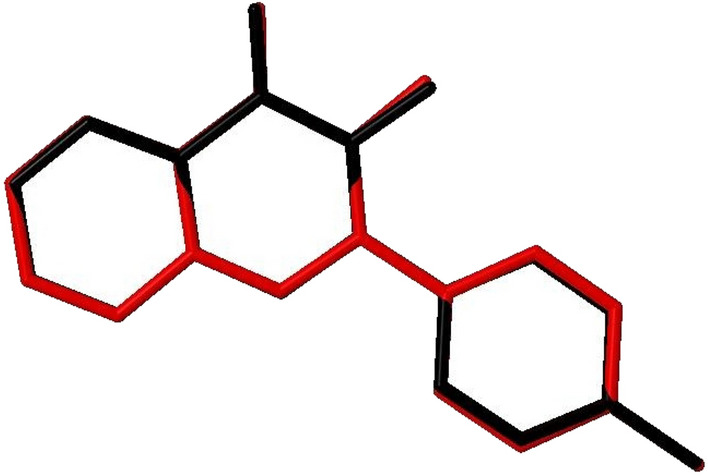
Superimposition atom-by-atom of the compound **12** calculated using DFT/6-31G(d, p) (red) over the X-ray structure (black). Hydrogen atoms omitted for clarity.

The ORTEP diagram for compound **12** in the solid state, which displayed the atomic numbering, is shown in Fig. 5. Also, detailed refinement details can be found in Table S1, while the bond lengths and angles can be found in Table S2. Notably, computed data produces from the isolated gaseous phase, while experimental data produces from the solid state. In Fig. 15, we see a remarkable agreement between the structural molecular geometry calculated by theoretical calculations and determined by X-ray analysis^50,51^.

#### FT-IR spectral analysis

**Figure 16:**
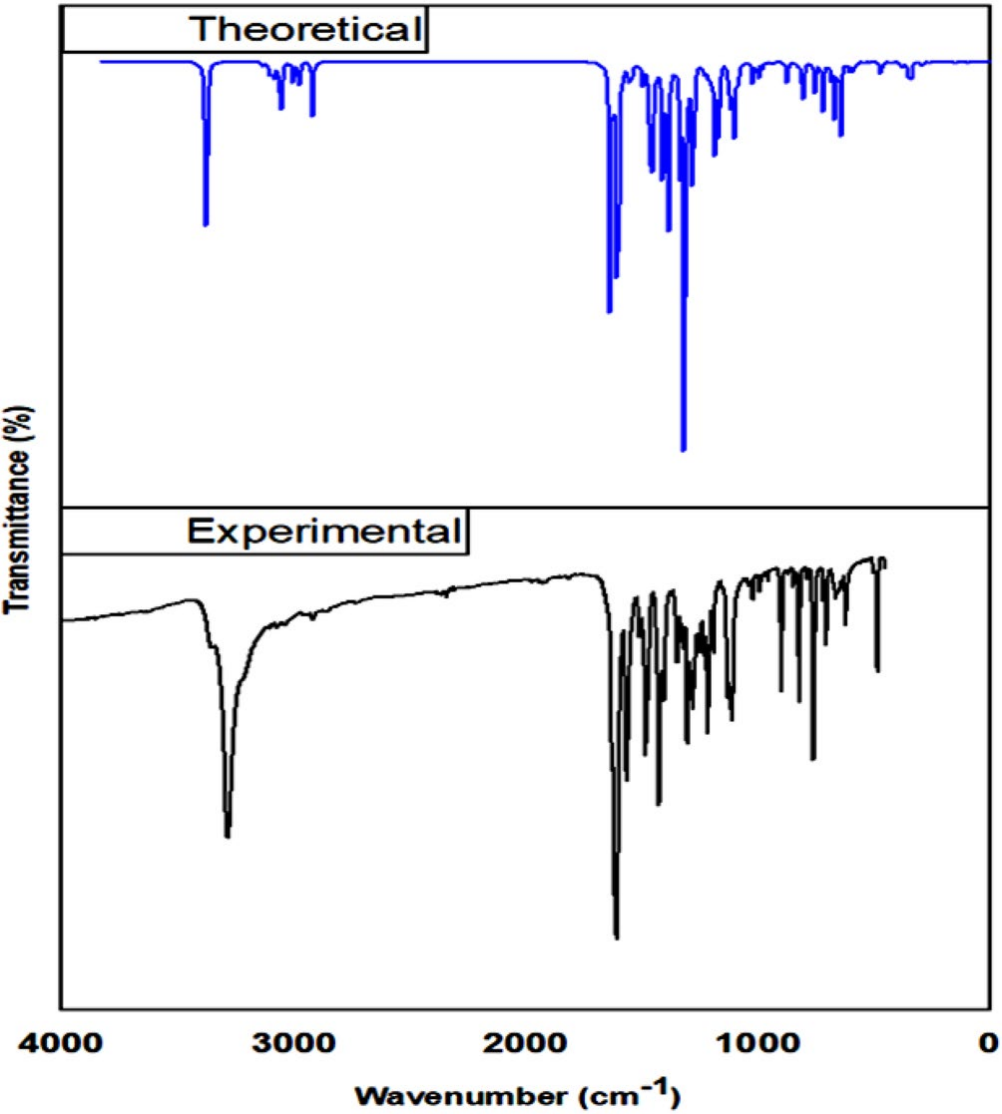
The FT-IR spectra of compound **12** experimental and theoretical calculated by B3LYP/6-31G(d,p) with scaling.

The molecule's geometry was taken directly from the experimental X-ray diffraction data and was not constrained by anything. Gauss-View molecular visualization program was used to assign vibrational bands^52^. Figure 16 shows the comparison between the observed and calculated vibrational frequencies of compound **12**. The IR spectrum of compound **12** reveals some characteristic bands associated with stretching vibrations of the O–H, C–H, and C=O groups. A key characteristic of the aromatic structure is the presence of C–H stretching vibrations in the region 2900–3150 cm^−1^. Experimentally, the C–H aromatic stretching mode was observed at 3107 cm^−1^ and computed at 3082 cm^−1^ for B3LYP. Additionally, the stretching vibration of the O–H group is experimentally observed at 3284 cm^−1^ and computed at 3380 cm^−1^. The experimental stretching vibration of C=O was observed at 1607 cm^−1^ and computed at 1638 cm^−1^. Discrepancies between the experimental and computed spectra arise from two possible causes, the first is the environment and the second is the fact that experimental values have anharmonic frequencies and calculated values have harmonic frequencies.

#### NMR correlation of compound 12

**Figure 17:**
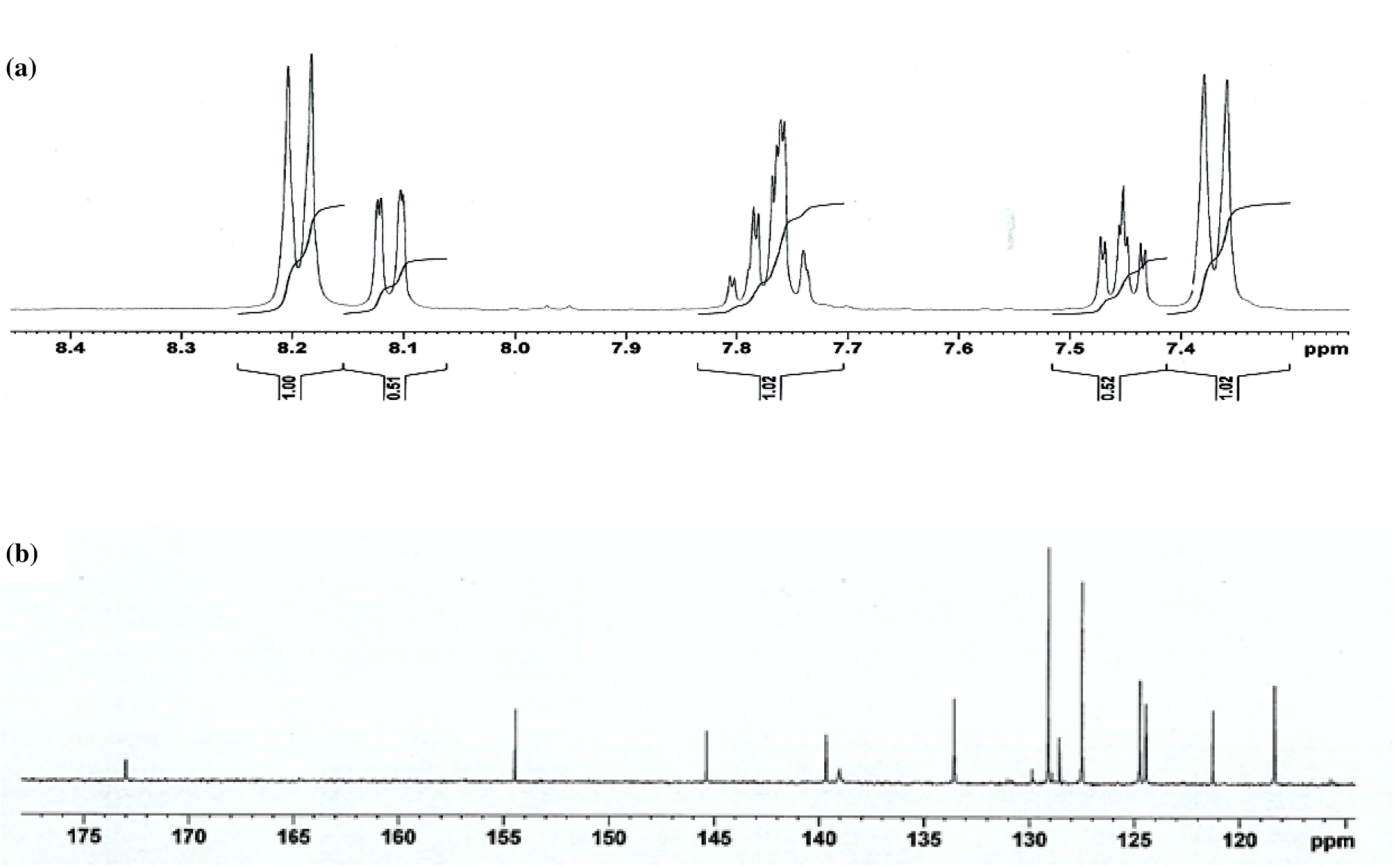
The experimental (**a**) ^1^H and (**b**) ^13^C NMR spectra of compound **12** in DMSO solution.

Currently, NMR has found its way into many fields of scientific research, medicine, and multiple industries. NMR spectra are characterized by chemical shifts that contain important information. Calculations of the proton and carbon chemical shifts were made using the GIAO method using the B3LYP/6-31G(d,p) basis set in DMSO solvent and then compared with the experimental chemical shift values^53,54^ as listed in Table 4. Figure 17 shows the experimental ^1^H and ^13^C NMR spectra of compound **12**, while Fig. 6 illustrates the positions of the atoms. According to the B3LYP/6-31G(d, p) method, the ^1^H chemical shift values are 2.359–8.948 ppm, whereas the experimental results are 2.392–8.184 ppm. Table 4 shows that the theoretical outcomes are consistent with the experimental data, with the exception of the hydroxyl proton (H2). Furthermore, the calculated ^13^C chemical shift values were observed at 12.81–157.33 ppm for B3LYP, whereas the experimental results were observed at 21.02–173.02 ppm. Table 4 also includes the calculated ^13^C NMR and experimental results. On the basis of Table 4, the theoretical ^1^H and ^13^C chemical shifts of compound **12** are generally in agreement with the experimental ^1^H and ^13^C shifts.

**Table 4:** Theoretical and experimental 1H and13C isotropic chemical shifts (with respect to TMS, all values in ppm) for compound **12**.

Atom	Experimental (ppm) (DMSO)	Calculated (ppm) B3LYP 6-31G(d,p) (DMSO)	Atom	Experimental (ppm) (DMSO)	Calculated (ppm) B3LYP 6-31G(d,p) (DMSO)
C1	21.02	12.81	H1c	2.392	2.3599
C15	118.36	104.56	H1A	2.392	2.4264
C11	121.28	107.97	H1B	2.392	2.7584
C13	133.57	110.27	H2	–	7.6015
C6	127.51	111.54	H3	7.359	7.6564
C12	124.74	112.72	H7	7.359	7.7063
C4	127.51	114.84	H13	7.437	7.8171
C7	129.11	115.18	H15	7.761	7.9080
C3	129.11	115.24	H14	7.765	8.0981
C5	139.04	115.65	H6	8.184	8.4958
C14	124.46	119.595	H12	8.101	8.5522
C9	145.37	126.92	H4	8.184	8.9489
C2	139.69	127.79			
C8	128.58	131.64			
C16	154.48	140.78			
C10	173.02	157.33

#### Molecular electrostatic potential (MEP) maps

**Figure 18:**
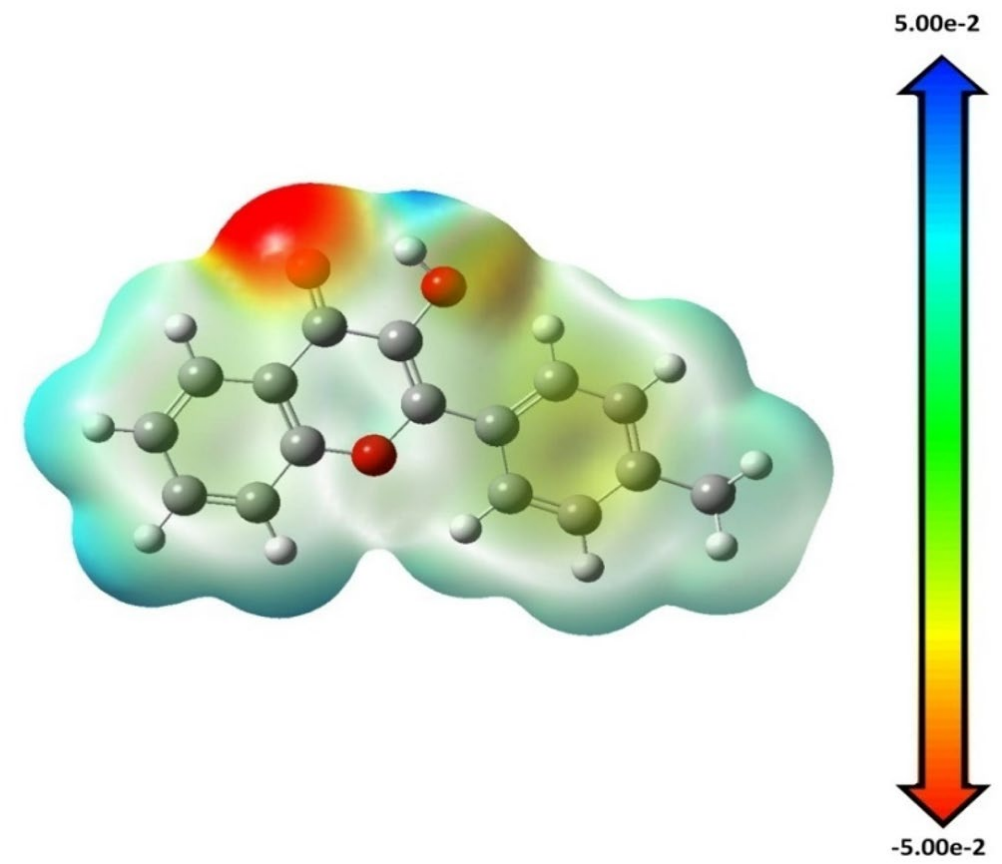
Molecular electrostatic potential map (MEP) calculated by B3LYP/6-31G(d,p) level.

MEP diagrams are used to predict biological processes and hydrogen bonding interactions as well as electrophilic and nucleophilic active sites^55^. It helps understand a molecule's relative polarity, anticipate its reactive site, and visualize its charge distribution in three dimensions. This is an excellent method for learning about molecular structure and physicochemical properties. MEP was computed based on B3LYP/6-31G(d,p) as displayed in Fig. 18 optimized geometry to determine the active sites for electrophilic and nucleophilic reactions in the compound. Generally, negative regions (red and yellow) show electrophilic reactivity, whereas positive regions (blue) show nucleophilic reactivity. Different colors indicate different electrostatic potentials at the surface, increasing in order from red to orange to yellow to green to blue. The map is highlighted between −0.05 a.u (deep red) and 0.05 a.u (deep blue) for the investigated compound, as shown in Fig. 18. Positive charges are mostly located on the nitrogen (N2) of the hydroxyl group, while negative charges are concentrated on the electron-negative atom O3 of the C=O.

## Experimental section

### General procedure

The Gallenkamp melting point instrument was used to measure the melting points. Thin-layer chromatography (TLC) was conducted on Polygram SIL G/UV254 TLC plates, and the results were visualized with ultraviolet light at 254 nm and 350 nm. In this study, the CEM Discover LabMate microwave apparatus (300 W, ChemDriver software; Matthews, NC) was used for microwave experiments. In closed vessels under pressure, microwave irradiated Pyrex tubes with caps were used to conduct the reactions. A Bruker DPX 400 superconducting NMR spectrometer was used to record the ^1^H and ^13^C nuclear magnetic resonance (NMR) spectra, and the IR spectra were measured with a Jasco Fourier transform/IR-6300 FT-IR spectrometer. The Elementar Vario MICRO Cube was used for the elemental analysis. Electron impact (EI) was used to determine mass analyses on a Thermo double-focusing sector (DFS) mass spectrometer. Varian Cary 5 spectrometer and a Shimadzu UV2600 spectrophotometer were used in UV − vis studies. Utilizing X-ray micro diffraction and single-crystal X-ray diffract meters, Rigaku D/MAX Rapid II and Bruker X8 Prospector were used to determine the X-ray crystal structure.

### Materials and reagents

1-(2-hydroxyphenyl)ethan-1-one (**1**), 4-methylbenzaldehyde (**2**) were purchased from Aldrich Chemical CO. In this study, all solvents were obtained from Aldrich.

### Preparation of 1-(2-hydroxyphenyl)-3-(*p*-tolyl)prop-2-en-1-one (3)

It was synthesized as reported in the literature^30,56^. To a mixture of 1-(2-hydroxyphenyl)ethan-1-one (**1**) (1.36 g, 10 mmol) and 4-methylbenzaldehyde (**2**) (10 mmol), a solution of 10% NaOH/H_2_O (20 ml) was added in portions to give blood-red solution. The reaction was stirred at room temperature overnight. Then the reaction mixture was acidified to pH 3 with concentrated HCl. The resulting yellow precipitate was filtered, washed with water and recrystallized from ethanol to afford the corresponding 1-(2-hydroxyphenyl)-3-(*p*-tolyl)prop-2-en-1-one (**3**). m.p: 118–119 °C. Yield, 80%, FT-IR (υmax, cm^−1^): 3089 (aromatic CH), 1637 (C=O), 1064 (C=C), 1563.3 (ArC–C–), 1200 (ArC–OH). 1H NMR (DMSO-*d6*, δ ppm): 2.39 (s, 3H, *H*3C), 6.09–7.01 (m, 2H, H-4′, 5′), 7.27 (d, 2H, *J* = 15.5 Hz, H-3′′, 5′′), 7.54–7.57 (dt, 1H,* J* = 8.4 Hz, H-3′), 7.79 (d, 2H,* J* = 8.4 Hz, H-2′′, 6′′), 7.80 (d, 1H, *J* = 15.6 Hz, H-2), 7.97 (d, 1H, *J* = 15.6 Hz, H-3), 8.24 (dd,1H, *J* = 8.4 Hz, H-6′), 12.59 (s, 1H, *H*O), ^13^C NMR (DMSO-*d6*):δ 21.09(*C*H3), 117.70(*C*H), 119.09(*C*H), 120.53(*C*H), 120.64(*C*H), 129.21(*C*H), 129.57(*C*H), 130.80(*C*H), 131.70(*C*H), 136.24(*C*H), 141.16(*C*H), 144.95(*C*H), 161.93(*C*–OH), 193.61(C=O), MS (*m/z*): 238 (M^+^, 100.0%), Anal. Calcd. for C16H14O2 (238.29): C, 80.65%; H, 5.92%. Found: C, 80.67%; H, 5.90%.

### Synthesis of 6-(2-hydroxyphenyl)-2-oxo-4-(*p*-tolyl)-1,2-dihydropyridine-3-carbonitrile (5)

The chalcone compound **3** (1 g, 4.2 mmol), ethyl cyanoacetate (0.474 g, 4.2 mmol), and ammonium acetate (1.28 g, 16.8 mmol) in absolute ethanol (4 ml) was irradiated utilizing microwave at 70 °C for 90 min. After cooling filter the formed precipitate and recrystallized from ethanol, Yellow crystals. Yield (0.2 g, 16%). M.p 290 °C. FT-IR (υ_max_, cm^−1^): 3174 (NH), 2217 (CN), 1637 (C=O), 1215 (ArC–OH). ^1^H NMR (DMSO-d6, δ ppm): 2.40 (s, 3H, CH3), 6.61 (brs, 1H, H-5), 6.91 (dt, 1H, J = 7.2 Hz, H-5′), 6.98 (d, 1H, J = 8.4 Hz, H-3′), 7.34–7.38 (m, 3H, H-3′′, 5′′, 4′), 7.60 (d, 2H, J = 8 Hz, H-2′′, 6′′, 6′), 10.59 (brs,1H, HN), 12.40 (s, 1H, HO), ^13^C NMR (DMSO-d6):δ 21.3 (CH3), 107.28 (C-5), 116.65 (C-1′), 119.09 (C-3), 119.34 (CN), 128.07 (C-3′), 128.26 (C-5′), 129.12 (C-6′), 129.34 (C-3′′, 5′′, 4′), 129.88 (C-1′′), 132.23 (C-2′′, 6′′), 133.28 (C-4′′), 140.27 (C-6), 155.78(C-4), 159.36 (C-2′), 161.48 (C-2), MS (*m/z*): 302 (M^+^, 100.0%), Anal. Calcd. for C19H14N2O2 (302.33): C, 75.48; H, 4.67; N, 9.27. Found: C, 75.40; H, 4.50; N, 9.30.

### Synthesis of 6'-acetyl-2''-hydroxy-4-methyl-1',6'-dihydro-[1,1':3',1''-terphenyl]-5'(2'H)-one (8)

A mixture of chalcone **3** (1 g, 4.2 mmol), acetyl acetone (0.42 g, 4.2 mmol) and NaOH (2 ml, 50%) in absolute ethanol (6 ml) was irradiated utilizing microwave at 25 °C for 90 min. The reaction mixture was poured into ice cold water. The resulting beige product was filtered, washed with water, dried and recrystallized with ethanol water mixture. Yield (0.2, 15%). Mp 85 °C. FT-IR (υ_max_, cm^−1^): 3107 (C–H Ar),1721 (C=O), 1625 (C=C). ^1^H NMR (DMSO-d6, δ ppm): 1.94 (s, 3H, H3C), 2.22 (s, 3H, H3C), 2.84–3.11 (m, 2H, H-4), 3.62–3.72 (m, 1H, H-5), 4.24 (d, 1H, J = 19.2 Hz, H-6), 6.39 (s, 1H, H-2), 6.74 (t, 1H, J = 12 Hz, H-5′), 6.82–6.87 (m, 2H, H-4′), 6.90 (d, 1H, J = 12.6 Hz, H-3′), 7.05–7.13 (m, 3H, H-3′′, 5′′, 6′), 7.23–7.30 (m, 2H, H-2′′, 6′′), 9.92 (s, 1H, OH), ^13^C NMR (DMSO-d6): δ 20.43 (CH3), 22.52 (CH3), 30.57 (C-4), 43.03 (C-5), 64.28 (C-6), 116.17 (C-3′), 119.10 (C-5′), 125.84 (C-1′), 127.21 (C-6′), 129.01 (C-2′′, 6′′), 130.74 (C-3′′, 5′′), 135.85 (C-4′), 139.01 (C-2), 155.20 (C-4′′), 159.93 (C-1′′), 179.71 (C-3), 191.60 (C-2′), 196.20 (C-1), 205.82 (CO). MS (*m/z*): 320 (M^+^, 45.0%), Anal. Calcd. for C_21_H_20_O_3_ (320.14): C, 78.73; H, 6.29. Found: C, 78.70; H, 6.30.

### Synthesis of 2-(*p*-tolyl)-1,2-dihydro-4H-thieno[2,3-*c*]chromen-4-one (10)

To a stirred solution of chalcone **3** (1 g, 4.2 mmol) and thioglycolic acid **9** (2.3 mL, 6.5 mmol) in methanol (5 mL) was added piperidine (2 ml) dropwise. The mixture was irradiated utilizing microwave at 70 °C for 90 min. pour the reaction mixture after completion into acidic ice water. Pale yellow precipitate formed (0.2 g, 16%). M p 154 °C. FT-IR (υ_max_, cm^−1^): 3028 (C–H Ar), 1711 (C=O), 1601 (C=C). ^1^H NMR (DMSO-d6, δ ppm): 2.29 (s, 3H, H3C), 3.72 (dd, 1H, J = 18 Hz, H-1A), 4.01 (dd, 1H, J = 18 Hz, H-1B), 5.36 (t, 1H, J = 6.4 Hz, H-2X), 7.17 (d, 2H, J = 7.8 Hz, H-3′, 5′), 7.36 (d, 2H, J = 9.6 Hz, H-2′, 6′), 7.39 (d, 1H, J = 7.8 Hz, H-8), 7.47 (d, 1H, J = 8.4 Hz, H-6), 7.57 (dt, 1H, J = 8.4 Hz, H-7), 7.64 (dd, 1H, J = 8.4 Hz, H-9), ^13^C NMR (DMSO-d6): δ 20.65 (CH3), 42.72 (C-2), 51.18 (C-1), 116.34 (C-6), 117.84 (C-9a), 124.85 (C-3a), 125.13 (C-8), 126.60 (C-9), 129.28 (C-2′, 6′), 130.49 (C-3′, 5′), 137.17 (C-7), 138.55 (C-1′), 147.09 (C-9b), 152.51 (C-5a), 156.03 (C-4). MS (*m/z*): 293 ((M-H)^+^, 100.0%), Anal. Calcd. for C_18_H_14_O_2_S (294.07): C, 73.44; H, 4.79. Found: C, 73.39; H, 4.77.

### Synthesis of 3-hydroxy-2-(*p*-tolyl)-4H-chromen-4-one (12)

The chalcone **3** (1 g, 4.2 mmol), was suspended in methanol (10 ml) and acetone (10 ml) and then NaOH (10%, 10 ml) and H_2_O_2_ (30%, 10 ml) was added at 4 °C. The mixture was stirred for 18 h at room temperature. It was poured on to cold 80 mL of 5 N HCl. The yellow solid was filtered, washed with water, dried and crystallized from methanol to afford compound with good yield (0.9 g, 85%). Mp 199 °C. FT-IR (υ_max_, cm^−1^): 3284 (OH), 3107 (C–H Ar), 1607 (C=O). ^1^H NMR (DMSO-d6, δ ppm): 2.39 (s, 3H, CH3), 7.35 (d, J = 12.6 Hz, 2H, H-3', 5'), 7.43–7.47 (m, 1H, H-7), 7.73–7.80 (m, 2H, H-8, 6), 8.10 (dd, J = 12.6 Hz, 1H, H-5), 8.18 (d, J = 12.6 Hz, 2H, H-2', H6'), ^13^C NMR (DMSO-d6): δ 21.02 (CH3), 118.36 (C-8), 121.28 (C-4a), 124.46 (C-7), 124.74 (C-5), 127.51 (C-2', 6'), 128.58 (C-2), 129.11 (C-3', 5'), 133.57 (C-6), 139.04 (C-1'), 139.69 (C-4'), 145.37 (C-3), 154.48 (C-8a), 173.02 (C-4). MS (*m/z*): 252 (M^+^, 100.0%), Anal. Calcd. for C_16_H_12_O_3_ (252.08): C, 76.18; H, 4.79. Found: C, 76.21; H, 4.72.

### Antioxidant activities

DPPH (2,2-diphenyl-1-picrylhydrazyl) radical scavenging activity. The scavenging activity of different heterocyclic compounds were determined using the free radical DPPH (2,2-diphenyl-1-picrylhydrazyl). Equal volumes of 100 µM DPPH chemical solution was mixed in methanol and added to different concentrations of the test compounds (0–200 µM/ml) in methanol and mixed well. The reaction mixture was incubated for 30 min at room temperature in the dark and was then measured at 520 nm. Plotting the percentage DPPH⋅ scavenging against concentration gave the standard curve and the percentage scavenging was calculated from the following equation:

$$\%\, scavenging\, =\, {\rm [(}Absorbance \, of \, blank \, - \, Absorbance \, of \, test {\rm )}/Absorbance \, of\, blank{\rm ]} \,\times \,100.$$

IC50 was obtained from a plot between concentration of test compounds and % scavenging. Ascorbic acid (vitamin C) was used as standard for comparison.

### Molecular docking simulation

The complex docking molecular analysis was enhanced with bond lengths in Å units using Moe software^57^. The minimization energies were then implemented to maintain the geometrical optimization and systematic investigations with an RMS gradient of 0.01 Å. Human peroxiredoxin 5 (PDB ID: 1HD2)^58^, also Crystal Structure of Klebsiella pneumoniae R204Q HpxO complexed with FAD (**PDBID**: 3RP8)^59^ The approval specified by E-conformation, total statistics, and related to amino acids surrounded by the binding compact of the protein^60,61^.

### Hirshfeld surface analysis

The topology analyses were performed using Crystal Explorer 17.5 program^48^.

### Computational studies

Molecular geometry was directly taken from the experimental outcomes of X-ray diffraction without any constraints. Density functional theory including Becke’s three-parameter hybrid functional using the LYP correlation functional (B3LYP) with the 6-31G(d, p) basis set via the Berny method^35,36,62^ were proceeded with the Gaussian 09W program^34^ For the optimized structure, harmonic vibrational frequencies were predicted at the same level of theory, and the resultant frequencies were scaled through 0.9663 for DFT^63^ The superimposition was performed using Olex2^64^. In order to investigate the reactive sites for flavone **12**, the molecular electrostatic potential was calculated using the B3LYP/6-31G(d, p) method. In dimethyl sulfoxide (DMSO), the ^13^C and ^1^H NMR chemical shifts were calculated using the gauge-invariant atomic orbital (GIAO) method^65–67^. The GIAO method is widely used in the computation of magnetic shielding tensors. This approach is often more accurate for the same basis set size because it allows the calculation of the absolute chemical shielding due to the electronic environment of individual nuclei. Based on the calculated absolute chemical shielding of TMS, the chemical shifts of ^1^H and ^13^C NMR have been converted into the TMS scale. These values are respectively 31.88 and 182.46 ppm for B3LYP/6–31+G(2d, p). Furthermore, the Mullikan atomic charges of flavanol **12** were calculated as well.

## Conclusion

In these studies, the chalcone showed higher activity towards the active methylene compounds using irradiant microwave. Most of the produced compounds showed excellent antioxidant activity due to their structures and the presence of more OH and C=O, which increases the action of ascorbic acid. All compounds were docked through different proteins to confirm the biological evaluation which showed the activity of compound 12 with two proteins with binding energy affinity −8.9 kcal/mol and −8.7 kcal/mol and the shortage bond length 1.31 Å and 2.67 Å; respectively which compatible with experimental results. Furthermore, the synthesized heterocyclic compounds were optimized through DFT basis set to determine their physical descriptors to confirm their biological results. Moreover, compound **12** its X-ray was correlated with theoretical results and Hirsh field analysis through bond length and angles and showed excellent correlated with FT-IR and NMR analysis.

## Supplementary Information

Supplementary Information.

## Data Availability

All data generated or analyzed during this study are included in this published article.
