# Supplementary material for: Microwave-assisted synthesis, antioxidant activity, docking simulation, and DFT analysis of different heterocyclic compounds
Source: Sci Rep. 2023 Mar 27;13:4999. doi: 10.1038/s41598-023-31995-w (PMC10042854; doi:10.1038/s41598-023-31995-w)
Supplement: Supplementary file 1 — Supplementary Information. [file 41598_2023_31995_MOESM1_ESM.docx]

***Supplementary material:***

**Table.S1**

**Table S1.** Selected Crystallographic data and refinement details for **12**.

| **Crystal sample** | **12** |
| --- | --- |
| Chemical formula | [C_16_H_12_O_3_](file:///G:\Mona\phd\experimental\Mona_MH13_230521%20_chemical_formula_sum) |
| *M*_r_ | [252.26](file:///G:\Mona\phd\experimental\Mona_MH13_230521%20_chemical_formula_weight) |
| Crystal system, space group | Monoclinic, [*P*2_1_/*n*](file:///G:\Mona\phd\experimental\Mona_MH13_230521%20_space_group_name_H-M_alt) |
| Temperature (K) | [296](file:///G:\Mona\phd\experimental\User-defined%20_cell_measurement_temperature) |
| *a*, *b*, *c* (Å) | [13.2851 (3)](file:///G:\Mona\phd\experimental\Mona_MH13_230521%20_cell_length_a), [5.1899 (1)](file:///G:\Mona\phd\experimental\Mona_MH13_230521%20_cell_length_b), [18.8025 (4)](file:///G:\Mona\phd\experimental\Mona_MH13_230521%20_cell_length_c) |
| β (°) | [109.232 (1)](file:///G:\Mona\phd\experimental\Mona_MH13_230521%20_cell_angle_beta) |
| *V* (Å^3^) | [1224.05 (5)](file:///G:\Mona\phd\experimental\Mona_MH13_230521%20_cell_volume) |
| *Z* | [4](file:///G:\Mona\phd\experimental\User-defined%20_cell_formula_units_Z) |
| Radiation type | [Cu *K*α](file:///C:\Users\h\Desktop\User-defined%20_diffrn_radiation_type) |
| µ (mm^−1^) | [0.77](file:///G:\Mona\phd\experimental\solve3%20_exptl_absorpt_coefficient_mu) |
| Crystal size (mm) | [0.22](file:///G:\Mona\phd\experimental\solve3%20_exptl_crystal_size_max) × [0.09](file:///G:\Mona\phd\experimental\solve3%20_exptl_crystal_size_mid) × [0.06](file:///G:\Mona\phd\experimental\solve3%20_exptl_crystal_size_min) |
| Diffractometer | [Bruker *APEX*-II CCD](file:///G:\Mona\phd\experimental\solve3%20_diffrn_measurement_device_type) |
| Absorption correction | [Multi-scan](file:///C:\Users\h\Desktop\User-defined%20_exptl_absorpt_correction_type)  [*SADABS2016*/2 - Bruker AXS area detector scaling and absorption correction](file:///G:\Mona\phd\experimental\solve3%20_exptl_absorpt_process_details) |
| *T*_min_, T_max_ | [0.45](file:///G:\Mona\phd\experimental\Mona_MH13_230521%20_exptl_absorpt_correction_T_min), [0.95](file:///G:\Mona\phd\experimental\Mona_MH13_230521%20_exptl_absorpt_correction_T_max) |
| No. of measured, independent & observed [*I*> 2σ(*I*)] reflections | [14232](file:///G:\Mona\phd\experimental\Mona_MH13_230521%20_diffrn_reflns_number), [2152](file:///G:\Mona\phd\experimental\Mona_MH13_230521%20_reflns_number_total), [1908](file:///G:\Mona\phd\experimental\Mona_MH13_230521%20_reflns_number_gt) |
| *R*_int_ | [0.063](file:///G:\Mona\phd\experimental\Mona_MH13_230521%20_diffrn_reflns_av_R_equivalents) |
| (sin θ/λ)_max_ (Å^−1^) | 0.596 |
| *R*[F^2^> 2σ(F^2^)], wR(F^2^), S | [0.064](file:///G:\Mona\phd\experimental\Mona_MH13_230521%20_refine_ls_R_factor_gt), [0.137](file:///G:\Mona\phd\experimental\Mona_MH13_230521%20_refine_ls_wR_factor_ref), [2.81](file:///G:\Mona\phd\experimental\Mona_MH13_230521%20_refine_ls_goodness_of_fit_ref) |
| No. of reflections | [2152](file:///G:\Mona\phd\experimental\Mona_MH13_230521%20_refine_ls_number_reflns) |
| No. of parameters | [174](file:///G:\Mona\phd\experimental\Mona_MH13_230521%20_refine_ls_number_parameters) |
| H-atom treatment | [Constrained](file:///C:\Users\talaz\AppData\Local\Packages\microsoft.windowscommunicationsapps_8wekyb3d8bbwe\LocalState\Files\S0\53\shelxl%20_refine_ls_hydrogen_treatment) |
| Δρ_max_, Δρ_min_ (e Å^−3^) | [0.23](file:///G:\Mona\phd\experimental\Mona_MH13_230521%20_refine_diff_density_max), [−0.38](file:///G:\Mona\phd\experimental\Mona_MH13_230521%20_refine_diff_density_min) |

**Table.S2**

**Table S2** Designated optimized bond length Å, and bond angle degrees dihedral angle degrees of Compound **12** utilized and B3LYP/6-31G(d):

| **Bond lengths** (Å) | | | **Bond angles (°)** | | |
| --- | --- | --- | --- | --- | --- |
|  | **X-Ray** | ***DFT/B3LYP*** |  | **X-Ray** | ***DFT/B3LYP*** |
| **O1-C8** | **1.373(2)** | 1.37687 | **C8-O1-C16** | 120.4(1) | 122.21259 |
| **O1-C16** | 1.364(2) | 1.3589 | **H2-O2-C9** | 109.5 | 102.62784 |
| **O2-H2** | 0.82 | 0.98453 | **O1-C8-C5** | 111.5(1) | 112.40096 |
| **O2-C9** | 1.351(2) | 1.35306 | **O1-C8-C9** | 120.7(1) | 118.96779 |
| **O3-C10** | 1.236(2) | 1.24292 | **C5-C8-C9** | 127.7(1) | 128.63122 |
| **C8-C5** | 1.469(2) | 1.4664 | **C16-C11-C10** | 119.2(1) | 118.43118 |
| **C8-C9** | 1.359(2) | 1.36977 | **C16-C11-C12** | 118.9(1) | 119.08109 |
| **C11-C16** | 1.380(2) | 1.40325 | **C10-C11-C12** | 121.9(1) | 122.48773 |
| **C11-C10** | 1.456(2) | 1.45528 | **O1-C16-C11** | 122.3(1) | 121.98624 |
| **C11-C12** | 1.404(2) | 1.40772 | **O1-C16-C15** | 116.3(1) | 116.9809 |
| **C16-C15** | 1.395(2) | 1.40126 | **C11-C16-C15** | 121.4(2) | 121.03286 |
| **C5-C4** | 1.393(2) | 1.40925 | **C8-C5-C4** | 122.4(1) | 121.98431 |
| **C5-C6** | 1.397(3) | 1.40853 | **C8-C5-C6** | 119.6(1) | 120.05032 |
| **C10-C9** | 1.446(2) | 1.46027 | **C4-C5-C6** | 117.9(1) | 117.96505 |
| **C4-H4** | 0.93 | 1.08109 | **O3-C10-C11** | 123.0(2) | 125.6024 |
| **C4-C3** | 1.382(2) | 1.39089 | **O3-C10-C9** | 121.3(2) | 118.37736 |
| **C3-H3** | 0.93 | 1.08714 | **C11-C10-C9** | 115.7(1) | 116.02024 |
| **C3-C2** | 1.386(3) | 1.40086 | **O2-C9-C8** | 120.2(1) | 124.08223 |
| **C12-H12** | 0.93 | 1.08482 | **O2-C9-C10** | 118.1(1) | 113.53582 |
| **C12-C13** | 1.377(2) | 1.38362 | **C8-C9-C10** | 121.7(1) | 122.38195 |
| **C6-H6** | 0.93 | 1.08289 | **C5-C4-H4** | 119.7 | 119.81566 |
| **C6-C7** | 1.384(3) | 1.38988 | **C5-C4-C3** | 120.6(2) | 120.44066 |
| **C2-C7** | 1.383(2) | 1.40112 | **H4-C4-C3** | 119.7 | 119.74324 |
| **C2-C1** | 1.508(3) | 1.50878 | **C4-C3-H3** | 119.1 | 118.89623 |
| **C13-H13** | 0.93 | 1.08518 | **C4-C3-C2** | 121.8(2) | 121.74296 |
| **C13-C14** | 1.392(3) | 1.40798 | **H3-C3-C2** | 119.1 | 119.36072 |
| **C15-H15** | 0.93 | 1.0845 | **C11-C12-H12** | 120 | 117.7674 |
| **C15-C14** | 1.370(3) | 1.38645 | **C11-C12-C13** | 120.0(2) | 120.22754 |
| **C7-H7** | 0.93 | 1.08713 | **H12-C12-C13** | 120 | 122.00506 |
| **C14-H14** | 0.93 | 1.0858 | **C5-C6-H6** | 119.8 | 119.56669 |
| **C1-H1A** | 0.96 | 1.09412 | **C5-C6-C7** | 120.5(2) | 120.85197 |
| **C1-H1B** | 0.96 | 1.09396 | **H6-C6-C7** | 119.8 | 119.58094 |
| **C1-H1C** | 0.96 | 1.09739 | **C3-C2-C7** | 117.4(2) | 117.61039 |
|  |  |  | **C3-C2-C1** | 121.1(2) | 121.16537 |
|  |  |  | **C7-C2-C1** | 121.6(2) | 121.21403 |
|  |  |  | **C12-C13-H13** | 120.1 | 120.28377 |
|  |  |  | **C12-C13-C14** | 119.9(2) | 119.87084 |
|  |  |  | **H13-C13-C14** | 120.1 | 119.8454 |
|  |  |  | **C16-C15-H15** | 120.7 | 119.22777 |
|  |  |  | **C16-C15-C14** | 118.7(2) | 118.84364 |
|  |  |  | **H15-C15-C14** | 120.7 | 121.9286 |
|  |  |  | **C6-C7-C2** | 121.8(2) | 121.38814 |
|  |  |  | **C6-C7-H7** | 119.1 | 119.15527 |
|  |  |  | **C2-C7-H7** | 119.1 | 119.45654 |
|  |  |  | **C13-C14-C15** | 121.1(2) | 120.94404 |
|  |  |  | **C13-C14-H14** | 119.4 | 119.75768 |
|  |  |  | **C15-C14-H14** | 119.4 | 119.29828 |
|  |  |  | **C2-C1-H1A** | 109.5 | 111.46725 |
|  |  |  | **C2-C1-H1B** | 109.5 | 111.51184 |
|  |  |  | **C2-C1-H1C** | 109.5 | 111.08237 |
|  |  |  | **H1A-C1-H1B** | 109.5 | 108.14144 |
|  |  |  | **H1A-C1-H1C** | 109.5 | 107.17907 |
|  |  |  | **H1B-C1-H1C** | 109.5 | 107.2478 |


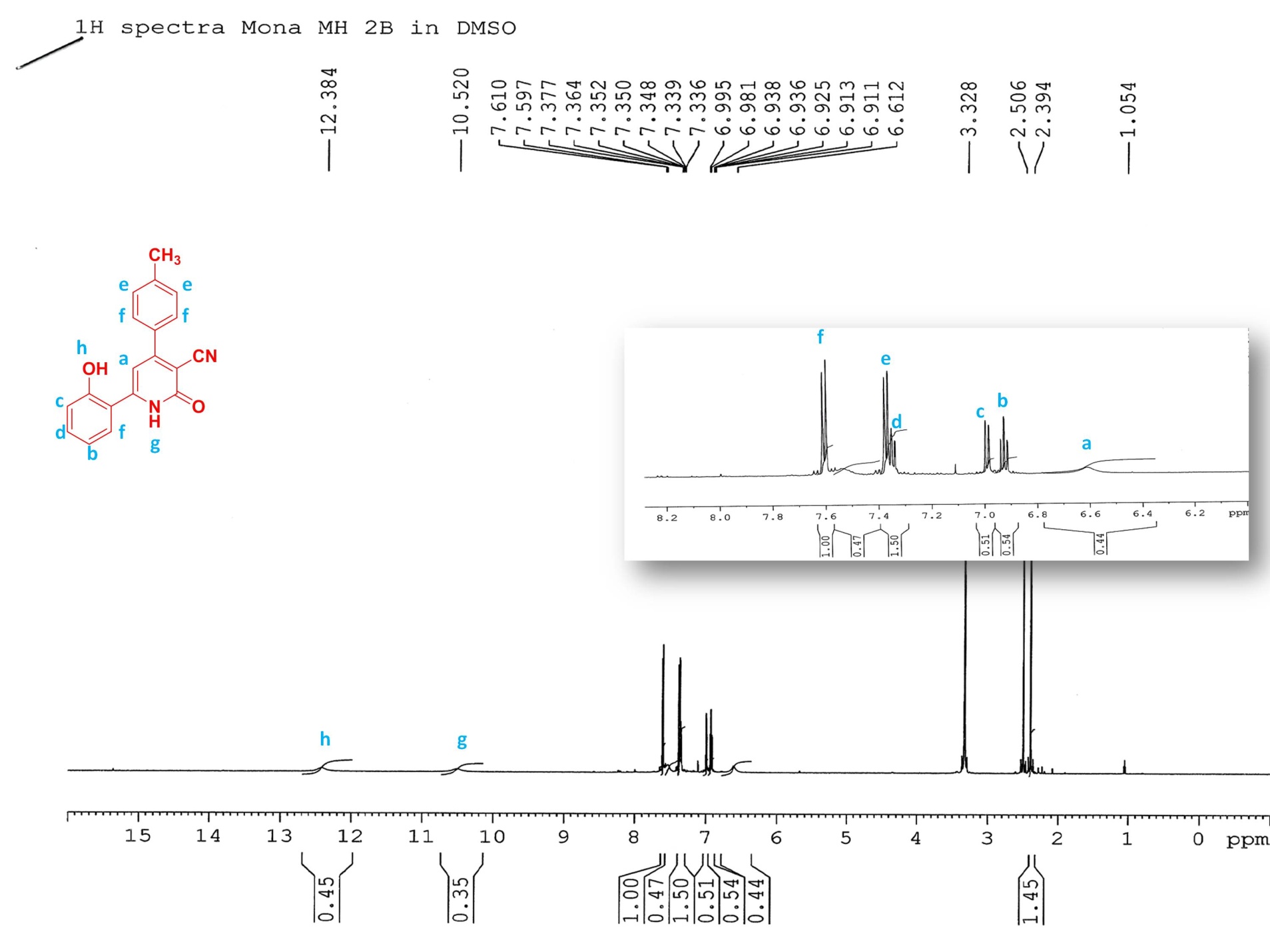


**Figure S1**. The ^1^H NMR spectrum of compound **5** (400 MHz, solvent DMSO- *d_6_*)

**
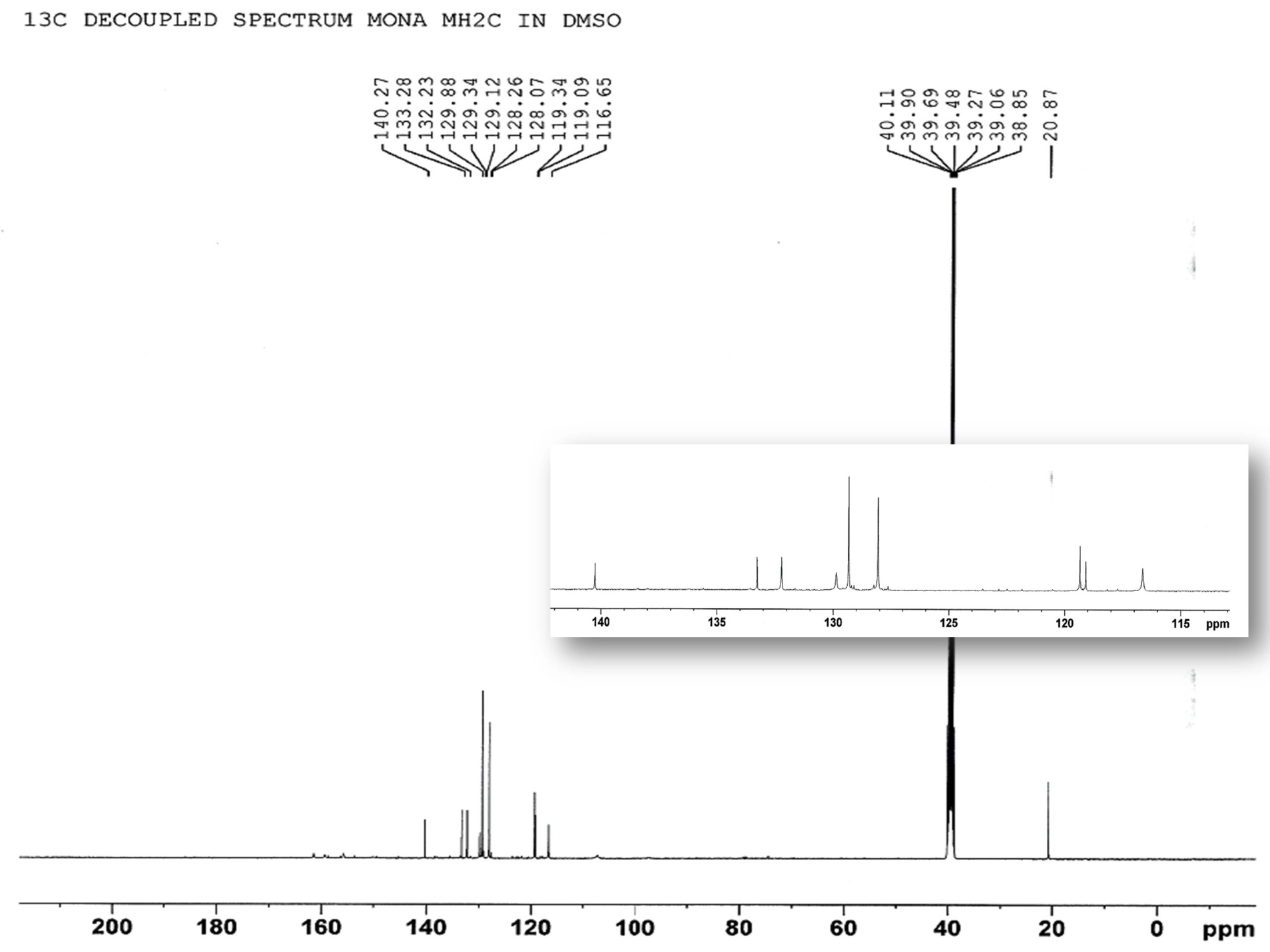
**

**Figure S2.** The ^13^C NMR spectrum of compound **5** (100 MHz, solvent DMSO- *d_6_*)


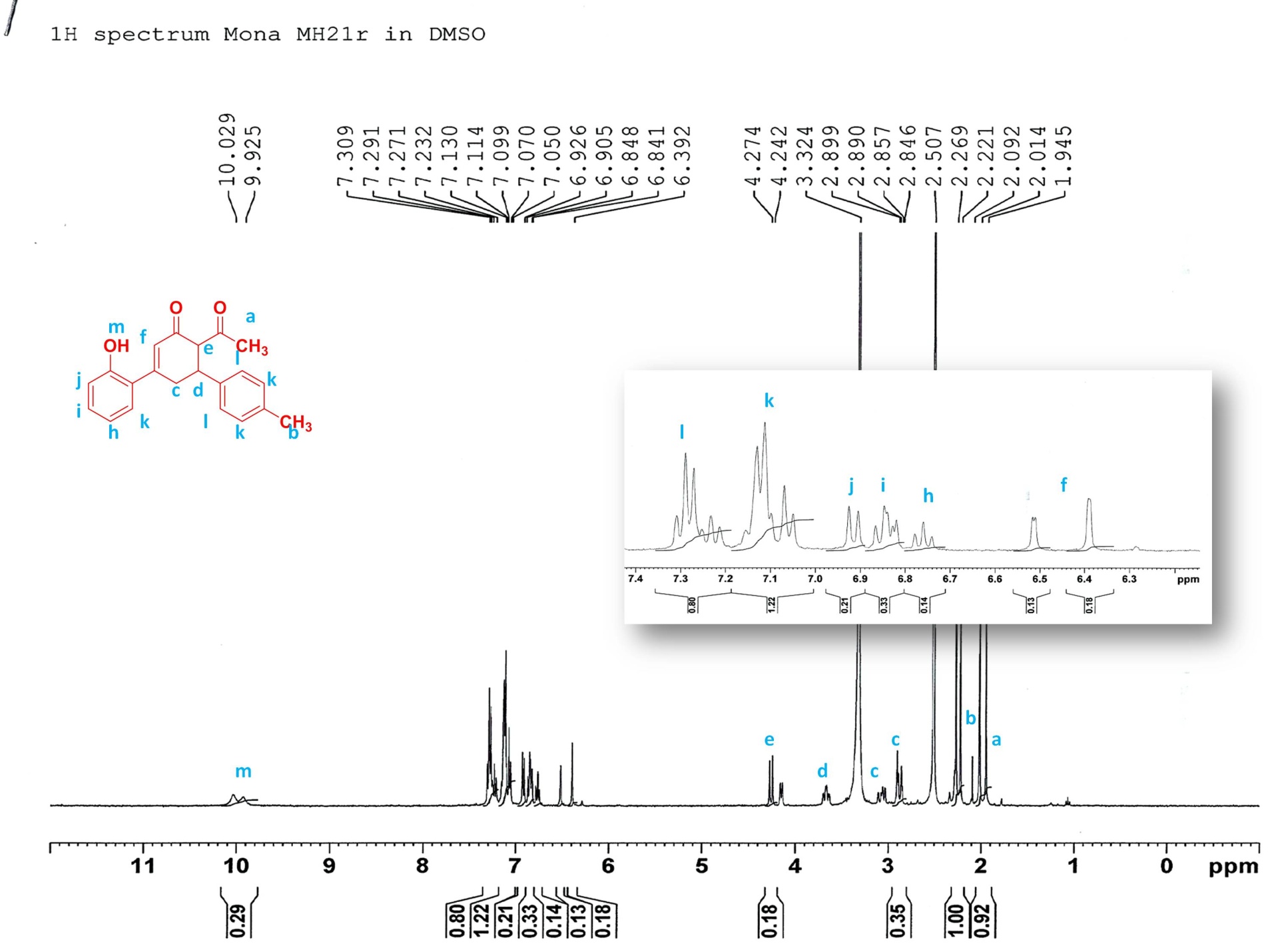


**Figure S3**. The ^1^H NMR spectrum of compound **8** (400 MHz, solvent DMSO- *d_6_*)


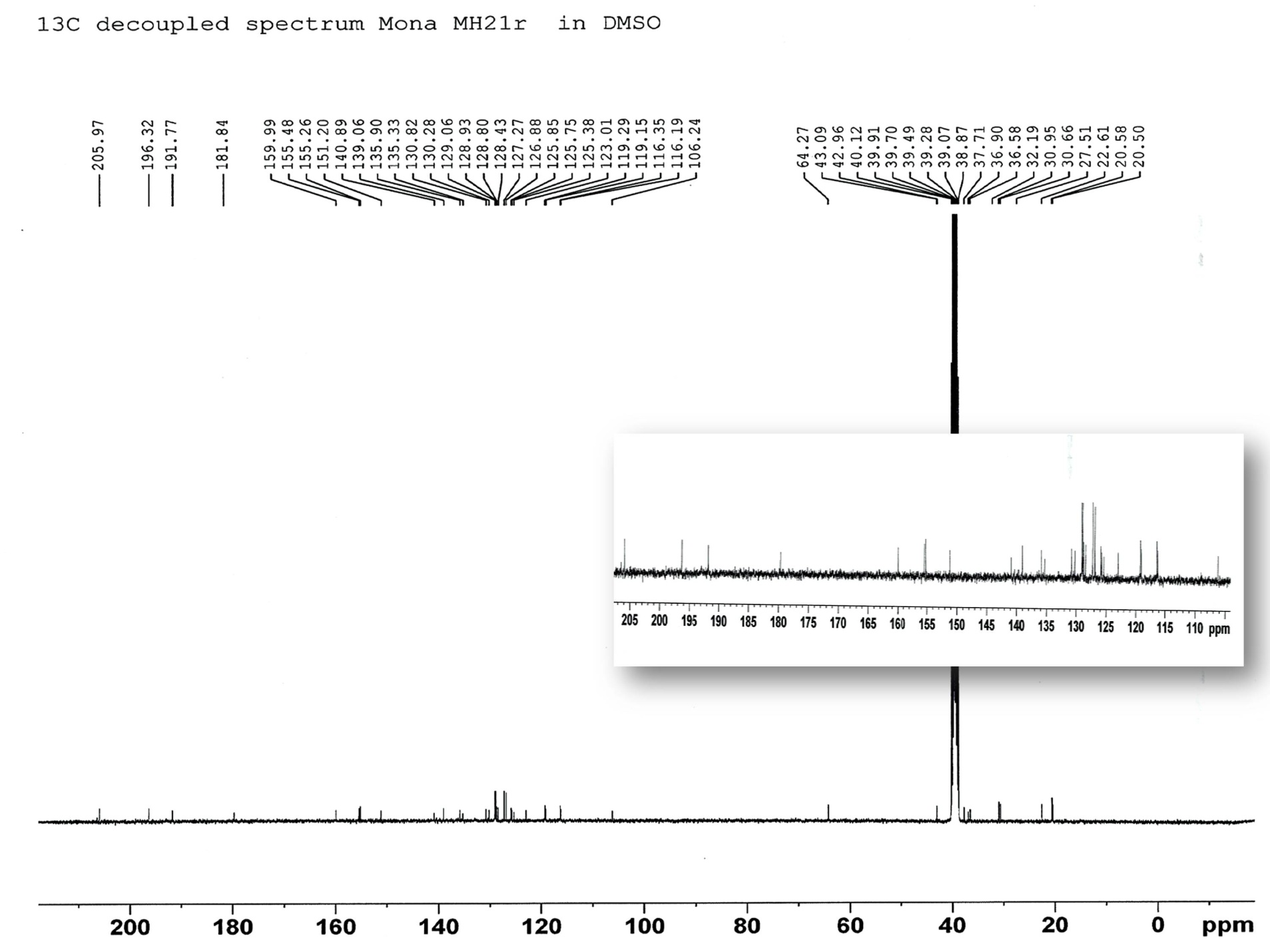


**Figure S4.** The ^13^C NMR spectrum of compound **8** (100 MHz, solvent DMSO- *d_6_*)


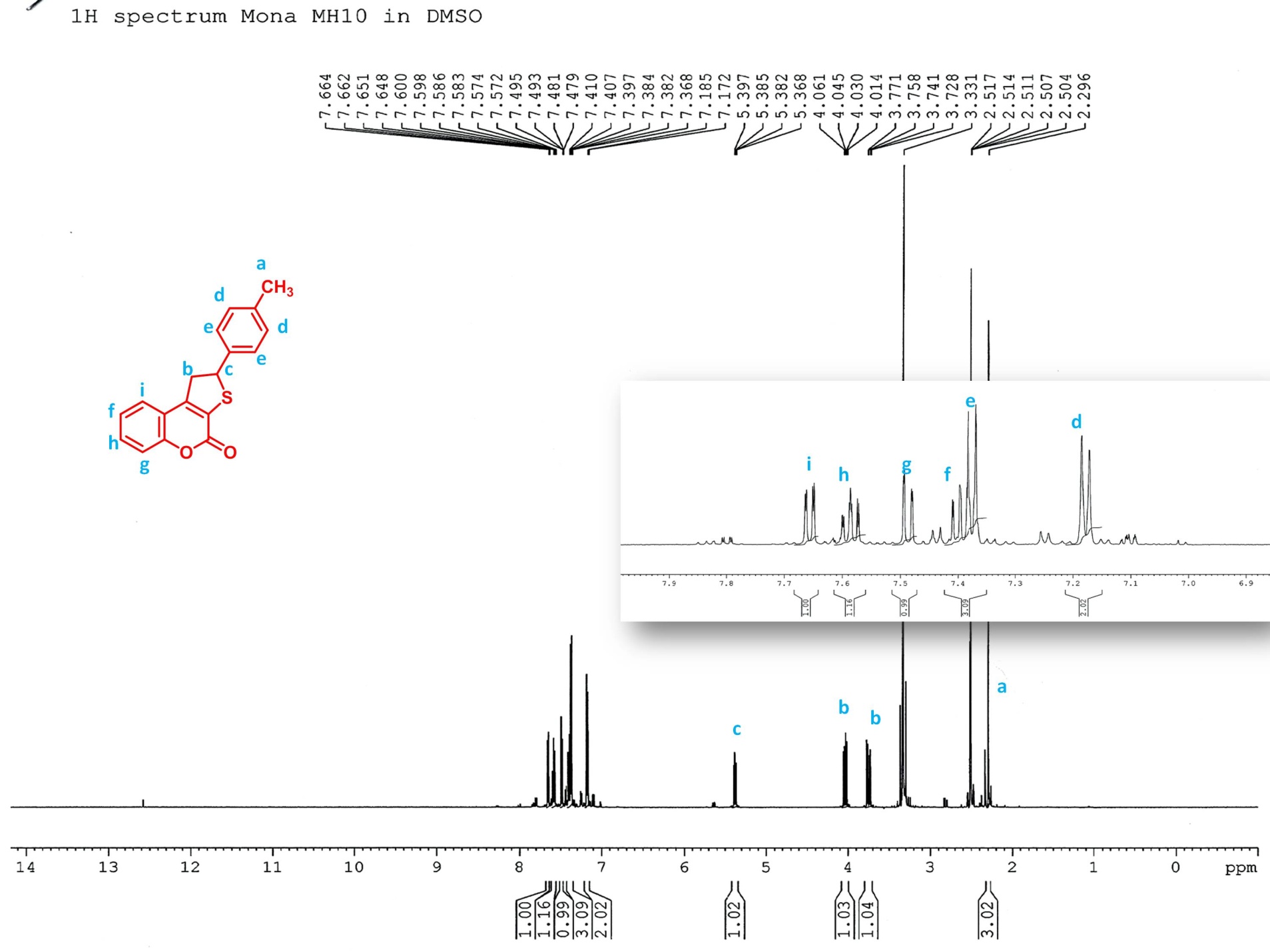


**Figure S5**. The ^1^H NMR spectrum of compound **10** (400 MHz, solvent DMSO- *d_6_*)


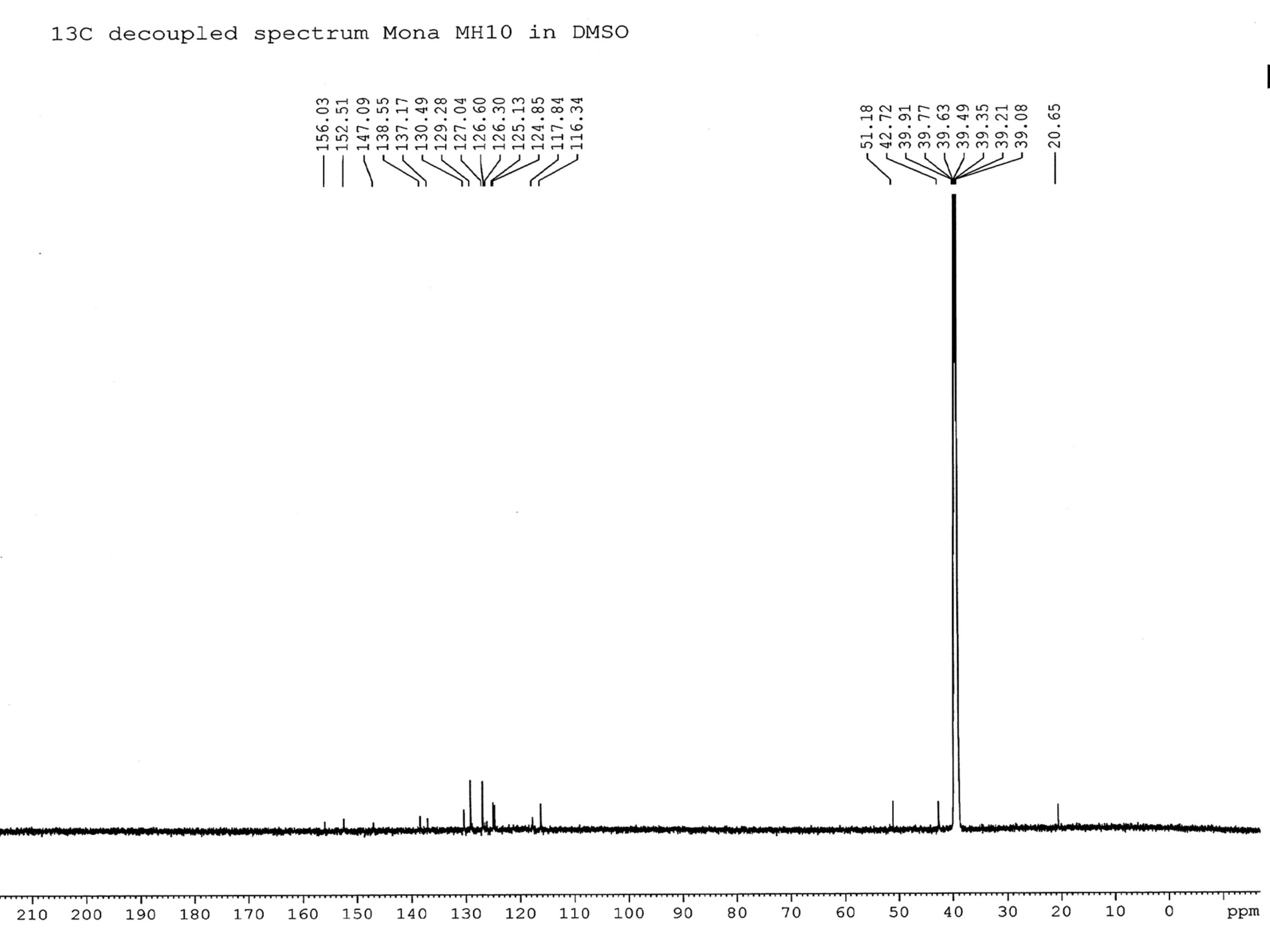


**Figure S6.** The ^13^C NMR spectrum of compound **10** (100 MHz, solvent DMSO- *d_6_*)


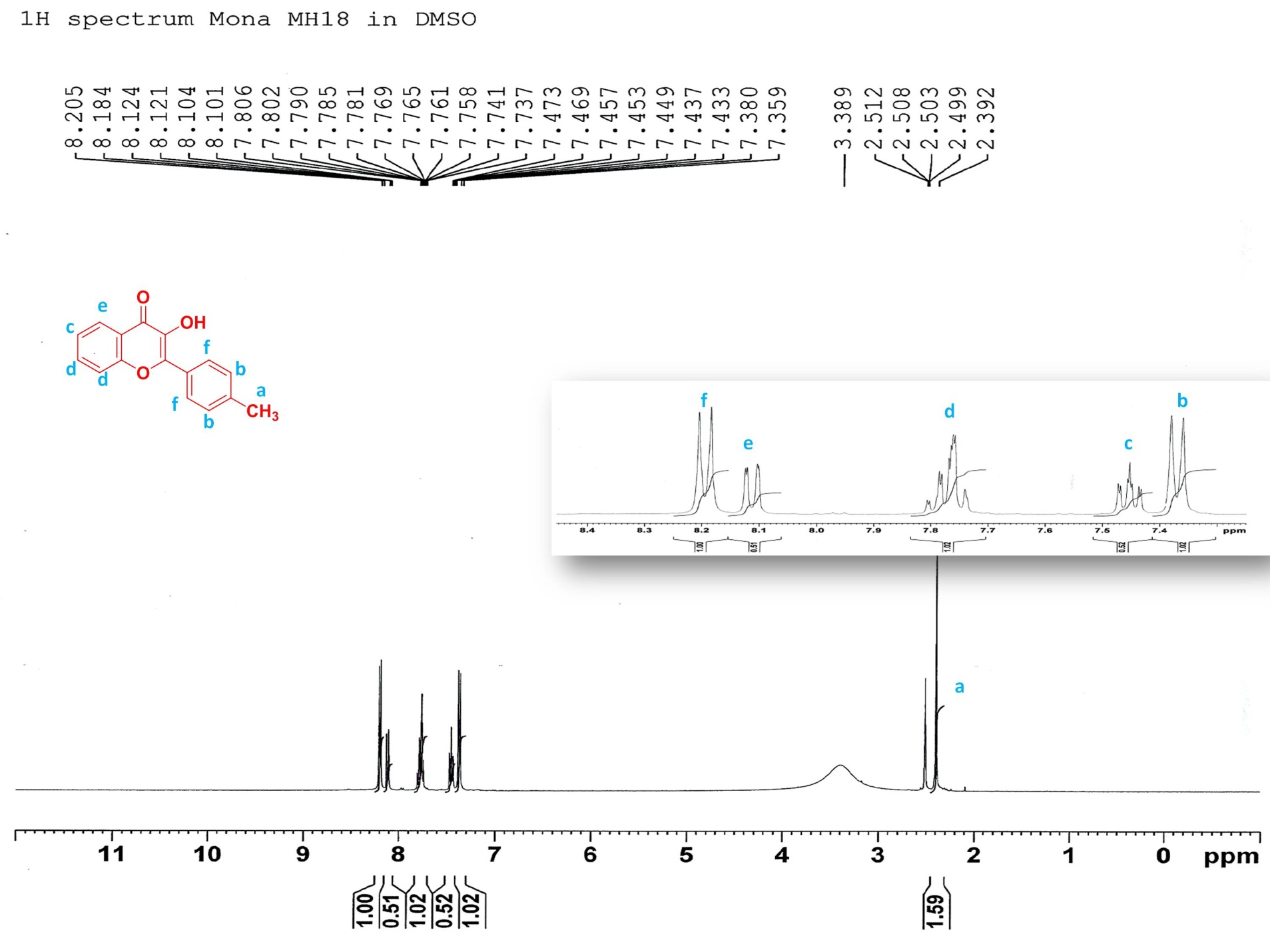


**Figure S7**. The ^1^H NMR spectrum of compound **12** (400 MHz, solvent DMSO- *d_6_*)


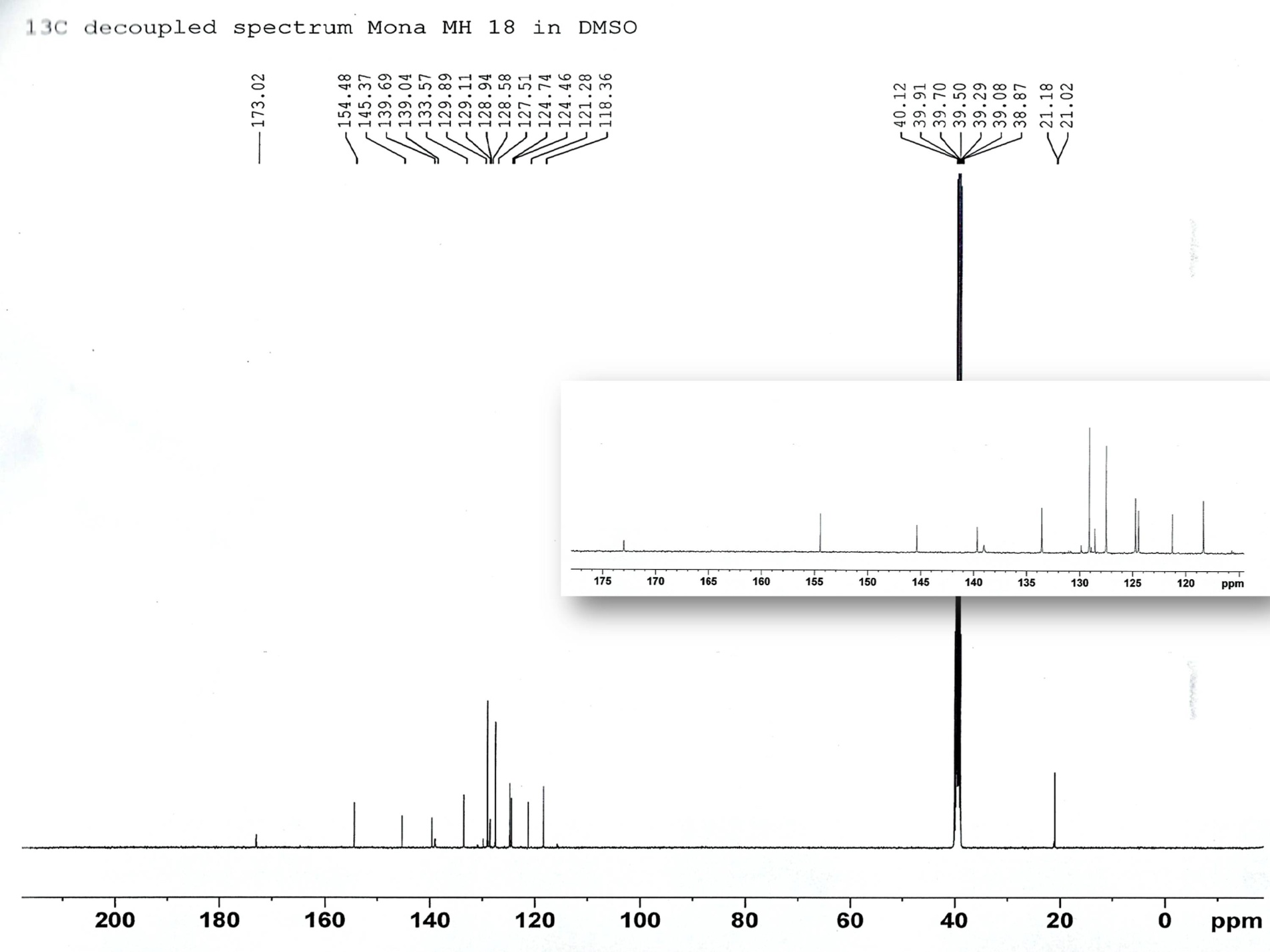


**Figure S8.** The ^13^C NMR spectrum of compound **12** (100 MHz, solvent DMSO- *d_6_*)
